# Bimetallic M_2_B Boride Nanoparticles: A Robust and Recyclable Platform for Dehydration‐Driven Condensation of Aldehydes

**DOI:** 10.1002/open.202500596

**Published:** 2025-12-16

**Authors:** Akram Ashouri, Arezu Moradi, Behzad Nasiri, Somayeh Pourian, Hossein Zamani, Fatemeh Rezaei, Amin Karimizadeh

**Affiliations:** ^1^ Department of Chemistry Faculty of Science University of Kurdistan Sanandaj Iran

**Keywords:** CoZnB‐NPs, dehydration condensation, heterogeneous catalysis, Lewis acid–base synergy, metal boride nanoparticles

## Abstract

Metal borides (MBs) emerge as a versatile class of nanomaterials, featuring high stability and bifunctional Lewis‐acidic/basic active sites. Despite these properties, their applications in C—N bond formation are less explored. Herein, we report the first systematic use of M_2_B‐type boride nanoparticles as efficient and magnetically recoverable catalysts for the condensation of aldehydes with *p*‐toluenesulfonamide under mild conditions. The synthesized CoZnB‐NPs exhibit partially oxidized and hydroxylated surfaces with Lewis‐acidic (B/Zn) and Lewis‐basic (Co) sites, where the synergistic interaction between Co and Zn centers facilitates charge transfer and stabilizes reactive intermediates. A series of mono‐ and bimetallic boride NPs are synthesized via aqueous NaBH_4_ reduction and comprehensively characterized by Fourier transform infrared spectroscopy (FT‐IR), field emission scanning electron microscopy (FE‐SEM), transmission electron microscopy (TEM), energy‐dispersive X‐ray spectroscopy (EDX), inductively coupled plasma optical emission spectroscopy (ICP‐OES), X‐ray diffraction (XRD), X‐ray photoelectron spectroscopy (XPS), and vibrating sample magnetometry (VSM). CoZnB‐NPs achieve yields of up to 93% within 1 h using an optimal catalyst loading of 3 mg in toluene, and demonstrate excellent magnetic recoverability and stability. This study highlights MB‐NPs as a surface‐engineered, high‐performance platform for selective C—N bond formation, providing insights into the design of bimetallic boride nanocatalysts for sustainable catalysis.

## Graphical abstract

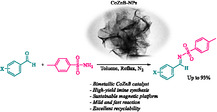

## 1 Introduction

Metal borides (MBs) are an emerging class of advanced materials distinguished by high hardness, excellent electrical conductivity, and high thermal stability [1, 2, 3]. These properties originate from strong covalent bonding between metal and boron atoms, leading to robust crystal lattices and tunable surface electronic structures [4]. Consequently, MBs have been widely explored in heterogeneous catalysis [5, 6], energy conversion [7], hydrogen production [8, 9], water splitting [10, 11], and electronic devices [12, 13]. MBs have exhibited high electrocatalytic activity and durability toward the hydrogen evolution (HER) and oxygen evolution (OER) reactions, even under harsh operating conditions [14, 15]. Furthermore, because MBs can be synthesized from abundant and low‐cost metals, they are regarded as economical and sustainable alternatives to noble‐metal‐based catalysts. The catalytic activity of MBs has been proposed to originate from their ability to activate hydrogen and to facilitate a wide range of elimination and hydrogenation processes [1, 16]. The coexistence of Lewis‐acidic and Lewis‐basic active sites on the nanoscale surface creates a favorable microenvironment. This arrangement facilitates complex multistep surface reactions. In addition, the incorporation of promoter metals such as Cr, Co, and Ni can effectively tune the electronic structure of the boride lattice, thereby modulating catalytic selectivity [16].

Nevertheless, the application of MBs in facilitating carbon–nitrogen (C—N) bond formation remains largely unexplored, offering a valuable opportunity for further catalytic development [17]. The condensation of carbonyl compounds with amines has long been regarded as one of the most straightforward and efficient routes to the synthesis of imines, serving as key intermediates in pharmaceutical, agrochemical, and materials chemistry [18, 19, 20, 21]. However, conventional methods have often been hindered by inefficient water removal, side reactions, and limited selectivity, thereby restricting their practical applications [22]. Therefore, considerable attention has been directed toward the development of heterogeneous catalysts capable of achieving simultaneous activation of electrophilic and nucleophilic centers and promoting selective and efficient dehydration [23, 24].

In this context, MB‐NPs, particularly those based on cobalt and zinc, have been considered as robust platforms for the selective synthesis of imines under mild conditions because of their bifunctional surface sites, high chemical and thermal stability, and tunable electronic structures [25, 26, 27]. In the present work, mono‐ and bimetallic M_2_B‐type boride NPs were synthesized and systematically investigated as nanocatalysts for the dehydration‐driven condensation of aldehydes with *p*‐toluenesulfonamide, producing sulfonyl aldimines. The catalytic performances of these materials were evaluated with particular attention to structure–activity relationships, operational stability, and mechanistic behavior. This study is believed to represent the first systematic investigation of MBs for such transformations, offering insights into their intrinsic dehydration function and potential as recyclable nanocatalysts for sustainable synthesis.

**SCHEME 1:**
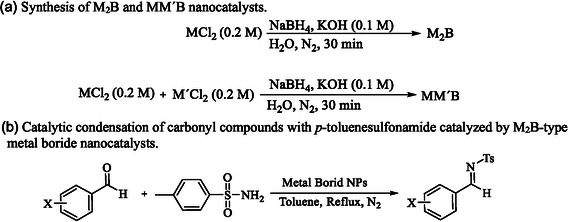
(a) Synthesis of M_2_B and MM′B nanocatalysts. (b) Catalytic condensation of carbonyl compounds with *p*‐toluenesulfonamide catalyzed by M_2_B‐type metal boride nanocatalysts.

MB‐NPs have been synthesized by various methods, each providing unique advantages for the control of morphology, phase composition, crystallinity, and particle size, which critically influence their catalytic behavior [27, 28]. Among these methods, aqueous reduction using NaBH_4_ has been widely employed due to its simplicity, reproducibility, and efficiency, typically yielding M_2_B‐type borides [25]. Based on this strategy, a series of mono‐ and bimetallic boride NPs were prepared under comparable conditions, allowing direct catalytic comparison, and their performances were evaluated in the condensation of carbonyl compounds with *p*‐toluenesulfonamide (Scheme 1).

## 2 Characterization of MB‐NPs

The structures of the synthesized MB‐NPs were comprehensively characterized using Fourier transform infrared (FT‐IR) spectroscopy, energy‐dispersive X‐ray spectroscopy (EDX), and field emission scanning electron microscopy (FE‐SEM) to confirm their phase purity, morphology, and textural properties (see Supporting Information). In addition, the bimetallic CoZnB‐NPs were further analyzed using transmission electron microscopy (TEM), BET, inductively coupled plasma optical emission spectroscopy (ICP‐OES), X‐ray diffraction (XRD), X‐ray photoelectron spectroscopy (XPS), and vibrating sample magnetometry (VSM), with the results discussed in the following subsections [25].

### 2.1 Characterization of CoZnB‐NPs

**FIGURE 1:**
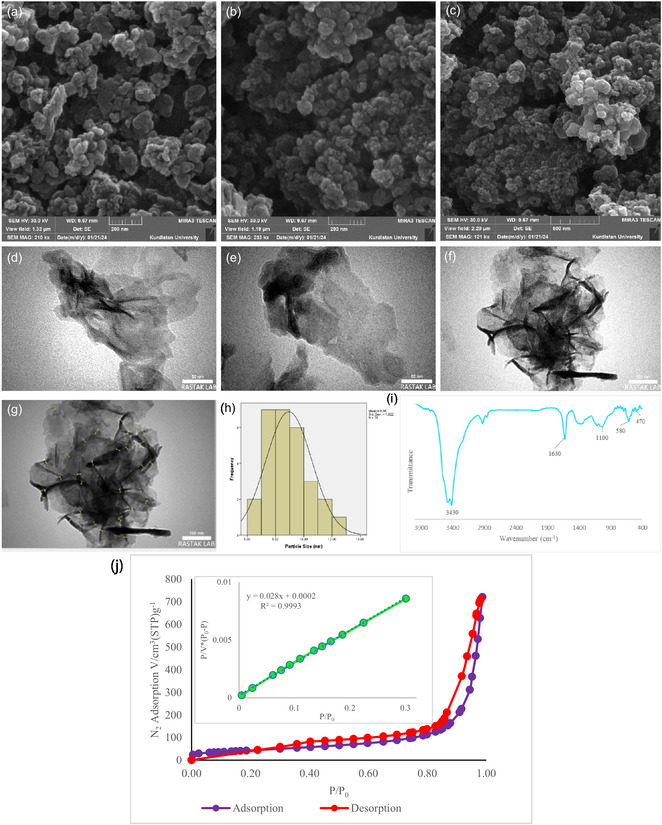
(a–c) The SEM images, (d–h) TEM images, (i) FT‐IR spectrum and (j) BET surface area and N_2_ adsorption plots of Co–Zn–B.

The FE‐SEM micrographs of the synthesized CoZnB‐NPs (Figure 1a–c) revealed a uniform granular morphology, characterized by well‐dispersed NPs with minimal agglomeration. This homogeneous distribution indicates effective synthesis and stable colloidal dispersion, supporting the high surface area and accessibility of catalytic sites observed in BET measurements (ABET = 154.3 m^2^/g, Figure 1j). TEM images (Figure 1d–h) further illustrated the nanoscale architecture. The Co–Zn–B nanocatalyst exhibited ultrathin, irregular nanosheets that self‐assembled into loosely packed, flower‐like aggregates. Individual nanosheets possess lateral dimensions of several tens of nanometers, with thicknesses confined to the nanometer scale. The intersheet voids created a porous texture that facilitated mass transport and exposure of active sites. Particle size analysis confirmed a narrow distribution, centered at 8.95 ± 1.62 nm (*n* = 28), reflecting precise control over nanostructure formation. The FT‐IR spectrum of the Zn–Co–B nanocatalyst (Figure 1i) exhibited a broad band around 3430 cm^−1^, which was assigned to O—H stretching of surface hydroxyl groups and adsorbed water. The peak at approximately 1630 cm^−1^ corresponds to the bending mode of physically adsorbed water molecules. The absorption band near 1100 cm^−1^ was attributed to B—O or B—O—B stretching vibrations, indicating partial oxidation of boron species and the formation of a thin surface oxide/hydroxide layer. In the low‐frequency region, bands at about 580 and 470 cm^−1^ are associated with Co–O and metal–boron (M–B) vibrations, respectively, confirming the formation of the metal boride framework with slight surface oxidation. Collectively, these findings indicate that the Zn–Co–B sample possessed an amorphous boride structure covered by a thin, hydroxylated, and partially oxidized surface layer. The nitrogen adsorption–desorption isotherm of the CoZnB sample, measured at 77 K, is presented in Figure 1j. The isotherm exhibits typical type IV behavior, as per the IUPAC classification, which is characteristic of mesoporous materials. The presence of a distinct H3‐type hysteresis loop in the relative pressure range of 0.8–1.0 suggests the formation of slit‐shaped pores due to the aggregation of plate‐like or sheet‐like particles. This results in an open, disordered mesoporous structure. The BET linear plot demonstrates an excellent linear fit within the relative pressure range of 0.05–0.30, with a high correlation coefficient (*R* ^2^ = 0.9993), confirming the accuracy of the data and the appropriate selection of the BET region. From the BET equation, the specific surface area of the CoZnB sample was calculated to be 154.3 m^2 ^g^−1^, with a monolayer adsorption volume (*V*_m_) of 35.45 cm^3^ (STP) g^−1^. The C constant value of 116.6 indicates a relatively strong interaction between nitrogen molecules and the surface of the adsorbent. The total pore volume at *p*/*p*_0_ = 0.987 was found to be 1.115 cm^3 ^g^−1^, and the average pore diameter was calculated to be 28.9 nm, confirming the presence of an open mesoporous network with a broad pore size distribution. Additionally, the BJH analysis (adsorption branch) revealed a primary pore size distribution peak centered around 9.2 nm, which is consistent with the characteristics of a type IV isotherm [29].

**FIGURE 2:**
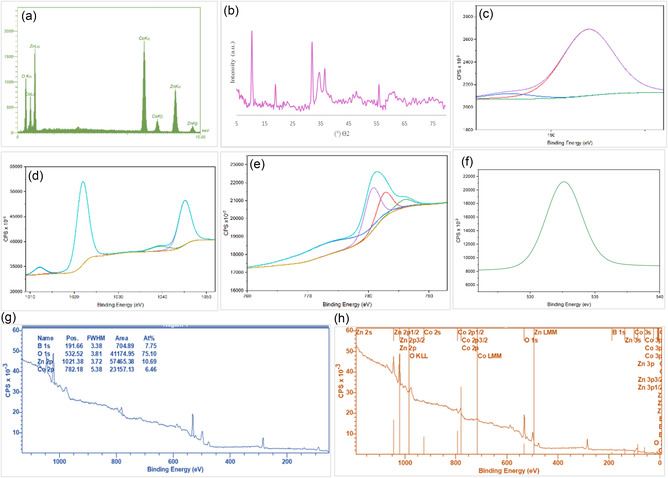
(a) The EDAX spectrum, (b) the XRD pattern, and (c–h) XPS spectra of B 1*s*, O 1*s*, Co 2*p*_3/2_, and Zn 2*p*_3/2_ of CoZnB‐NPs.

**FIGURE 3:**
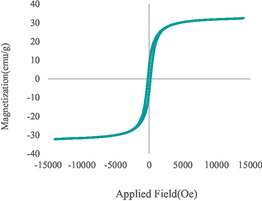
The VSM curve of CoZnB‐NPs.

To evaluate elemental composition and confirm successful bimetallic incorporation, EDX and ICP‐OES analyses were performed. EDX confirmed the presence of both cobalt and zinc in the CoZnB‐NPs, showing an approximate 1:1 Co:Zn atomic ratio at the analyzed surface (Figure 2a). Bulk quantification by ICP‐OES revealed Co and Zn contents of 32.87 wt% and 30.68 wt%, respectively. These values correspond to a bulk atomic ratio of Co:Zn 1.19:1. Boron was detected at 7.20 wt%, confirming its successful incorporation into the boride matrix. The near‐equimolar Co/Zn ratio verified successful bimetallic incorporation, while the minor difference between surface and bulk compositions was attributed to surface oxidation, as confirmed by XPS. The XRD pattern of the synthesized Co–Zn–B NPs (Figure 2b) exhibited a broad diffuse halo centered at 2*θ *= 30–50°, characteristic of an amorphous or poorly crystalline transition‐metal boride framework. The incorporation of boron atoms into the metallic lattice disrupted long‐range ordering and significantly reduced crystallinity, in line with previous reports on chemically reduced Co– and Ni–borides. Superimposed on this broad background, several weak reflections were observed at 36.5°, 45.1°, and 48.0°, which were indexed to orthorhombic Co_2_B (JCPDS No. 03‐065‐0418) and cubic Co (JCPDS No. 15‐0806) phases. Additional minor peaks at 55.9° and 61.5° correspond to ZnB (JCPDS No. 01‐077‐2376) and Co–Zn alloy phases, confirming partial alloy formation and efficient incorporation of Zn into the Co–B framework. A very weak diffraction feature at 75.2° was attributed to slight surface oxidation during synthesis or air exposure; however, this oxide phase remains negligible compared with the dominant boride matrix. The average crystallite size, estimated from the most intense reflection using the Scherrer equation, was approximately 9.6 nm, consistent with the nanoscale morphology observed in the TEM images. The calculated lattice strain for the CoZnB‐NPs was also found to be 1.06%, indicating a slight distortion of the crystal lattice compared to the ideal structure. These results collectively indicate that the as‐prepared CoZnB‐NPs possess a hybrid amorphous–crystalline structure, in which the amorphous boride matrix provides abundant unsaturated active sites, while the embedded nanocrystalline Co–Zn–B domains enhance structural integrity, thereby benefiting their catalytic performance [30]. The surface chemical composition and oxidation states of the CoZnB nanocatalyst were analyzed by XPS (Figure 2c‐h). The survey spectrum confirmed the presence of Co, Zn, B, and O, verifying successful bimetallic boride formation. The Zn 2*p* spectrum displays well‐defined doublet peaks at binding energies of 1021.3 eV (Zn 2*p*_3/2_) and 1045.5 eV (Zn 2*p*_1/2_), characteristic of Zn^2+^ species in ZnO, and with negligible contribution from minor Zn(OH)_2_ phases. The Co 2*p* region (782.2 eV) exhibits complex spin–orbit doublets with pronounced shake‐up satellite peaks, indicating the coexistence of Co^2+^ and Co^3+^ oxidation states, consistent with mixed CoO/Co_3_O_4_ or Co(OH)_2_ species on the surface. The B 1*s* spectrum reveals two distinct components: a minor peak at 189 eV attributed to metallic boron (B°) and a dominant one centered at 191.6 eV corresponding to oxidized boron species (B—O bonds), suggesting partial surface oxidation and passivation of the boride framework. The O 1*s* region can be deconvoluted into two contributions at 532.5 eV, assigned to lattice oxygen (O^2−^), surface hydroxyl groups (–OH), as well as adsorbed oxygen/water species. The coexistence of mixed‐valence Co cations (Co^2+^/Co^3+^), Zn^2+^‐oxide species, and oxidized boron indicates a surface composed of a thin, redox‐active oxide/hydroxide layer covering an amorphous boride core. Such a surface configuration can facilitate efficient electron transfer during catalytic reactions and enhance both stability and reusability under redox conditions. The magnetic properties of the CoZnB‐NPs were investigated at room temperature using VSM, and the resulting magnetization curve is shown in Figure 3. The nanocatalyst exhibits soft magnetic behavior with a narrow hysteresis loop, indicative of low coercivity (Hc ~ 118 Oe) and near‐superparamagnetic behavior, consistent with the nanoscale dimensions and the predominantly amorphous Co–Zn–B core. The saturation magnetization (*M*_s_) was measured at 31.2 emu g^−1^, which is higher than previously reported values for similar cobalt‐based borides, reflecting the effective incorporation of Co and Zn into the boride framework. This hybrid magnetic structure, combining high *M*_s_, low Hc, and dual magnetic domains, facilitates rapid magnetic separation and catalyst recovery while maintaining colloidal stability during operation [25].

## 3 Results and Discussion

The catalytic performance of the examined MB‐NPs was comprehensively investigated in the condensation of aldehydes with *p*‐toluenesulfonamide, revealing composition‐ and loading‐dependent trends. Initially, a series of monometallic boride NPs were synthesized, and their catalytic activities were evaluated under identical conditions [30]. As summarized in Table 1, the reactions proceeded efficiently in the presence of MB‐NPs under refluxing toluene, affording the corresponding aromatic aldimines in consistently high yields. Monometallic boride NPs such as Fe_2_B, Ni_2_B, and Zr_2_B exhibited moderate catalytic activity, with observed yields of 56%, 53%, and 60%, respectively, owing to their limited Lewis acid–base surface properties and the absence of synergistic interactions (Entries 1–3). Zn_2_B showed slightly higher efficiency (65%) owing to its stronger Lewis acidity, which facilitates carbonyl activation, while Cu_2_B afforded 71% yield through enhanced carbonyl polarization (Entries 4 and 5). Among the monometallic catalysts, Co_2_B displayed superior performance, yielding 78%, reflecting its Lewis‐basic sites and improved substrate activation (Entry 6). In the case of bimetallic borides, synergistic electronic and structural effects played a decisive role. CoZnB‐NPs achieved the highest yield of 89% (Entry 14). This enhancement results from cooperative electronic and structural interactions between cobalt and zinc within the bimetallic framework, which promote more efficient substrate activation and transition‐state stabilization. Other bimetallic combinations demonstrated intermediate yields within 65–80%, depending on the degree of surface synergism and metal dispersion (Entries 7–13). The catalytic performance was also investigated as a function of catalyst loading (1, 3, 5, and 10 mg of CoZnB‐NPs). Changing the catalyst loading to either 1 mg or 10 mg resulted in lower yields. The decline at lower loading is attributed to the limited availability of active sites, whereas at higher loading, particle aggregation hampers substrate accessibility. The optimal catalytic performance was obtained with 3 mg of CoZnB‐NPs, achieving the highest yield within the shortest reaction time of 1 h (Entries 15–17).

**TABLE 1:** Evaluation of metal boride NPs on the reaction.

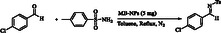			
Entry^a^	Catalyst	Time, h	Yield, %^b^
1	Fe_2_B	3	56
2	Ni_2_B	3	53
3	Zr_2_B	3	60
4	Zn_2_B	2.5	65
5	Cu_2_B	2	71
6	Co_2_B	2	78
7	CuFeB	2.5	70
8	CuCoB	2	78
9	CuZnB	2.5	70
10	CuZrB	3	63
11	CuNiB	2.5	65
12	CoNiB	2.5	62
13	CoZrB	2.5	69
14	CoZnB	1.5	89
15	CoZnB (10 mg)	2.5	75
16^c^	CoZnB (3 mg)	1	93
17	CoZnB (1 mg)	2.5	71

a Reaction conditions: Aldehyde (0.1 mmol), *p*‐toluenesulfonamide (0.1 mmol), dry toluene (1 mL), metal boride NPs (5 mg).

b Isolated Yields.

c For 1 h.

To examine the influence of solvent polarity and solvation properties on substrate solubility and reaction kinetics, the effect of different solvents on the condensation reaction was systematically studied (Table 2). The results revealed that polar protic solvents such as ethanol and methanol suppressed the reaction, giving no conversion in ethanol and only a trace yield in methanol (Entries 1 and 2), likely due to competitive hydrogen bonding and strong adsorption of solvent molecules on the catalyst surface. Similarly, aprotic polar solvents including THF, chloroform, dichloromethane, and acetonitrile (Entries 3–6) afforded only trace product formation, reflecting either insufficient stabilization of intermediates or poor solubility of the substrates. In contrast, aromatic solvents displayed more favorable performance, as *o*‐xylene and *p*‐xylene enabled moderate conversion (Entries 7 and 8), while dry toluene provided the highest yield (93%) in the presence of CoZnB‐NPs (Entry 9), due to its balanced polarity, effective solvation of reactants, and compatibility with the catalytic surface. Finally, a control experiment at room temperature (Entry 10) gave only 10% yield after 5 h, highlighting the essential role of thermal energy in accelerating dehydration and imine formation.

**TABLE 2:** Evaluation of solvent effect on the synthesis of sulfonyl imine.

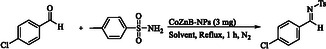		
Entry^a^	Solvents	Yield, %^b^
1	Ethanol	No product
2	Methanol	trace
3	Tetrahydrofuran	trace
4	Chloroform	trace
5	Acetonitrile	trace
6	Dichloromethane	trace
7	*o*‐Xylene	45
8	*p*‐Xylene	47
9	Toluene	93
10^c^	Toluene	10

a Reaction conditions: Aldehyde (0.1 mmol), *p*‐toluenesulfonamide (0.1 mmol), (1 mL).

b Isolated Yields.

c Room temperature.

After optimizing the reaction parameters, the scope of the protocol was systematically examined using a series of aromatic aldehydes containing both electron‐donating and electron‐withdrawing substituents (Table 3). In all cases, the reactions proceeded efficiently, yielding the corresponding imine derivatives in good to excellent conversions. The electronic nature of the substituents had minimal impact on product formation, highlighting the broad applicability and operational stability of the catalytic system.

**TABLE 3:** Synthesis of aromatic aldimine derivatives.

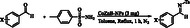				
Entry^a^	X	Yield, %^b^	M.p., °C	M.p. *Lit.*, °C
1	H	88	80–81	79–82 [18]
2	*p‐*Cl	93	165–168	175–179 [18]
3	*o‐*Cl	89	131–133	134–137 [18]
4	*m‐*Cl	91	90–91	84–88 [18]
5	*p‐*Br	97	189–192	190–193 [18]
6	*p‐*F	94	127–29	129–130 [18]
7	*p‐*CF_3_	98	150–154	154–156 [18]
8	*p‐*NO_2_	95	202–204	203–207 [18]
9	*p‐*OMe	86	109–111	111–114 [18]
10	*p‐*Me	90	126–128	129–130 [18]
11	*m‐*NO_2_	83	144–145	143–145 [19]
12	2,4‐dimetoxy	89	121–123	124–126 [31]
13	*o‐*OH	90	122–124	125 [19]

a Reaction conditions: Aldehyde (0.1 mmol), *p*‐toluenesulfonamide (0.1 mmol), (1 mL), CoZnB‐NPs (3 mg).

b Isolated Yields.

**SCHEME 2:**
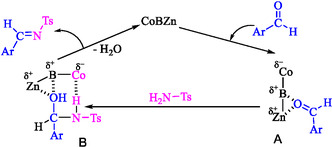
Proposed mechanism.

In the proposed reaction mechanism, the carbonyl group of the aldehyde is initially coordinated to the Lewis‐acidic sites of boron and zinc (δ^+^). This interaction polarizes the C=O bond and enhances the electrophilicity of the carbonyl carbon (**A**). Subsequently, the amine nucleophile attacks the activated carbon, generating a hydroxyl–amine intermediate (**B**). At this stage, the hydroxyl group is stabilized through coordination with the B/Zn centers, while cobalt sites (δ^−^), with higher electron density, abstract the proton, thereby facilitating the dehydration step. The removal of water results in the formation of the C=N double bond and the stable imine product. Finally, the proton adsorbed on cobalt is released, regenerating the active sites of the catalyst for the next cycle. This bifunctional acid–base synergy, where B/Zn acts as Lewis‐acidic centers and Co functions as a Lewis‐basic center, accounts for the excellent catalytic efficiency of CoZnB‐NPs toward imine formation (Scheme 2) [32].

## 4 Experimental Procedures

### 4.1 Synthesis of M_2_B‐NPs

The corresponding metal salt (4 mmol for single‐MBs or 2 mmol each for bimetallic borides) was dissolved in 20 mL of distilled water at 5°C and stirred under a nitrogen atmosphere for 10 min. Subsequently, an aqueous solution of NaBH_4_ (5 mmol, 190 mg in 10 mL) and KOH (2 mmol, 112 mg in 20 mL) was added dropwise over 30 min at 25°C. The resulting precipitate was separated magnetically when possible; otherwise, it was collected by filtration, washed several times with distilled water to remove residual impurities, and dried in an oven at 110°C for 2 h under vacuum [33].

### 4.2 General Procedure for the Preparation of Aromatic Aldimines in the Presence of CoZnB‐NPs

In a Schlenk tube under a nitrogen atmosphere, aldehyde (0.1 mmol), *p*‐toluenesulfonamide (0.1 mmol, 17 mg), and CoZnB‐NPs (3 mg) were added to dry toluene (1 mL). The mixture was heated under reflux for 1 h. Upon completion, the reaction mixture was cooled to room temperature, after which the magnetic catalyst was separated using an external magnet, and the solvent was removed under reduced pressure. The resulting solid was washed with *n*‐hexane and recrystallized from toluene. The structure of the synthesized imines was confirmed by melting point determination and ^1^H NMR spectroscopy (CDCl_3_, 500 MHz) [18].

### 4.3 Example Compounds

#### 4.3.1 N‐(3‐chlorobenzylidene)‐4‐methylbenzenesulfonamide

m.p. 90–91°C, ^1^H NMR (600 MHz, CDCl_3_) δ 8.09 (s, 1 H), 7.99 (d, *J* = 7.7 Hz, 1 H), 7.81 (d, *J* = 8.5 Hz, 2 H), 7.59 (d, *J* = 8.1 Hz, 1 H), 7.43 (t, *J* = 8.1 Hz, 1 H), 7.32 (d, *J* = 8.2 Hz, 2 H), 7.26 (s, 1 H), 2.43 (s, 3 H).

#### 4.3.2 N‐(2‐chlorobenzylidene)‐4‐methylbenzenesulfonamide

m.p. 131°C‐133°C, ^1^H NMR (600 MHz, CDCl_3_) δ 9.70 (s, 1 H), 7.97 (d, *J* = 8.1 Hz, 2 H), 7.85 (d, *J* = 8.2 Hz, 1 H), 6.81 (d, *J* = 8.1 Hz, 1 H), 6.75 (d, *J* = 8.1 Hz, 2 H),6.67 (d, *J* = 8.2 Hz, 2 H), 6.57 (d, *J* = 7.7 Hz, 1 H), 2.43 (s, 3 H).

#### 4.3.3 N‐(4‐chlorobenzylidene)‐4‐methylbenzenesulfonamide

m.p. 165°C‐168°C, ^1^H NMR (500 MHz, CDCl_3_) δ 8.99 (s, 1 H), 7.85–7.89 (m, *J* = 8.5 Hz, *J* = 8.3 Hz, 4 H), 7.46 (d, *J* = 8.5 Hz, 2 H), 7.35 (d, *J* = 8.2 Hz, 2 H), 2.43 (s, 3 H).

#### 4.3.4 N‐(4‐methylbenzylidene)‐4‐methyl‐ benzenesulfonamide

m.p. 126‐128°C, ^1^H NMR (500 MHz, CDCl_3_) δ 8.99 (s, 1 H), 7.89 (d, *J* = 7.5 Hz, 2 H), 7.82 (d, *J* = 7.4 Hz, 2 H), 7.34 (d, *J* = 7.49 Hz, 2 H), 7.29 (d, *J* = 7.43 Hz, 2 H) 2.43 (s, 3 H) 2.42 (s, 3 H).

#### 4.3.5 N‐(4‐Methoxybenzylidene)‐4‐methyl‐benzenesulfonamide

m.p. 109‐111°C, ^1^H NMR (500 MHz, CDCl_3_) δ 8.94 (s, 1 H), 7.87 (d, *J* = 8 Hz, 4 H), 7.32 (d, *J* = 7.7 Hz, 2 H), 6.96 (d, *J* = 8.2 Hz, 2 H), 3.87 (s, 3 H), 2.42 (s, 3 H).

#### 4.3.6 N‐(4‐(trifluoromethyl)‐4‐methyl‐benzenesulfonamide

m.p. 150‐154°C, ^1^H NMR (600 MHz, CDCl_3_) δ 9.07 (s, 1 H), 8.04 (d, *J* = 8 Hz, 2 H), 7.91 (d, *J* = 8.0 Hz, 2 H), 7.75 (d, *J* = 8.5 Hz, 2 H), 7.37 (d, *J* = 8.0 Hz, 2 H), 2.45 (s, 3 H).

### 4.4 Recycling Process of the Nanomagnetic Catalyst

**FIGURE 4:**
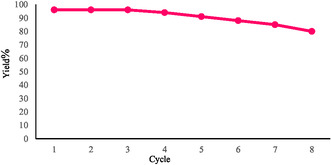
Correlation between catalyst reusability and the yield of product.

The recyclability of the CoZnB‐NPs was evaluated over eight consecutive catalytic cycles to assess their long‐term operational stability. After each reaction, the nanocatalyst was readily separated using an external magnet, washed thoroughly with distilled water and ethanol, and then dried under vacuum before reuse. As illustrated in Figure 4, the CoZnB‐NPs retained most of their catalytic activity over successive runs, showing only a slight decrease in product yield after the eighth cycle, thereby confirming their excellent stability and reusability. The catalytic performance was further analyzed by calculating the turnover number (TON) and turnover frequency (TOF) based on the total cobalt and zinc content obtained from EDX and ICP–OES analyses. CO chemisorption experiments indicated that 4.83% of the surface Co atoms were catalytically active under the reaction conditions. Using this experimentally determined active‐site fraction, the surface‐normalized TON and TOF were both estimated to be approximately 2418 h^−1^, evidencing the high intrinsic catalytic activity and durability of the CoZnB nanocatalyst. Postreaction structural analyses of the recovered CoZnB‐NPs after the eighth cycle—performed by FT‐IR and FE‐SEM (see Supporting Information)—revealed no significant change in morphology, surface functionalities, or magnetic properties, confirming the structural integrity and outstanding reusability of the material.

## 5 Conclusion

In summary, a series of mono‐ and bimetallic M_2_B‐type MB‐NPs were successfully synthesized and systematically evaluated as efficient nanocatalysts for the selective condensation of aldehydes with *p*‐toluenesulfonamide. Among them, CoZnB‐NPs exhibited the highest catalytic activity, affording up to 93% yield within short reaction times under refluxing toluene, thereby demonstrating excellent catalytic efficiency and reproducibility. The superior performance is attributed to the strong synergistic interaction between Co and Zn centers, which enhances charge transfer, promotes electronic coupling, and stabilizes reactive intermediates. Surface characterization revealed that CoZnB‐NPs possess a partially oxidized, hydroxylated surface enriched with Lewis‐acidic (B/Zn) and Lewis‐basic (Co) sites. This bifunctional surface architecture efficiently facilitates substrate adsorption, proton abstraction, and dehydration during imine formation, highlighting the critical role of surface composition and oxidation states in governing catalytic activity. The catalyst also displayed remarkable structural robustness and recyclability, maintaining its morphology, surface chemistry, and magnetic properties over at least eight consecutive reaction cycles, as confirmed by FE‐SEM and FT‐IR analyses. CO chemisorption analysis further determined that 4.83% of surface Co atoms is catalytically active. Using this experimentally obtained value, the surface‐normalized TON and TOF were both determined to be approximately 2418 h^−1^, confirming the high intrinsic catalytic efficiency and long‐term durability of CoZnB‐NPs. These findings highlight how controlled surface design, bimetallic synergy, and tunable Lewis acid–base functionality in M_2_B‐type borides can enable sustainable and high‐performance nanocatalysts for organic transformations involving C—N bond formation.

## Supporting Information

Additional supporting information can be found online in the Supporting Information Section. **Supporting Fig. S‐1:** FT‐IR of Fe_2_B. **Supporting Fig. S‐2:** FE‐SEM of Fe_2_B. **Supporting Fig. S‐3:** EDX of Fe_2_B. **Supporting Fig. S‐4:** FT‐IR of Cu_2_B. **Supporting Fig. S‐5:** FE‐SEM of Cu_2_B. **Supporting Fig. S‐6:** EDX of Cu_2_B. **Supporting Fig. S‐7:** FT‐IR of Co_2_B. **Supporting Fig. S‐8:** FE‐SEM of Co_2_B. **Supporting Fig. S‐9:** EDX of Co_2_B. **Supporting Fig. S‐10:** FT‐IR of Zn2B. **Supporting Fig. S‐11:** FT‐IR of Zr_2_B. **Supporting Fig. S‐12:** FT‐IR of CoZrB. **Supporting Fig. S‐13:** FE‐SEM of CoZrB. **Supporting Fig. S‐14:** EDX of CoZrB. **Supporting Fig. S‐15:** FT‐IR of CoNiB. **Supporting Fig. S‐16:** FE‐SEM of CoNiB. **Supporting Fig. S‐17:** EDX of CoNiZrB. **Supporting Fig. S‐18:** FT‐IR of CuCoB. **Supporting Fig. S‐19:** FE‐SEM of CuCoB. **Supporting Fig. S‐20:** EDX of CuCoB. **Supporting Fig. S‐21:** XRD of CuCoB. **Supporting Fig. S‐22:** FT‐IR of CuZnB. **Supporting Fig. S‐23:** FT‐IR of CuNiB. **Supporting Fig. S‐24:** FT‐IR of CuZrB. **Supporting Fig. S‐25:** FT‐IR of FeCuB. **Supporting Fig. S‐26:** CO chemisorption isotherm of the CoZnB NPs. **Supporting Fig. S‐27:** FT‐IR spectrum of of CoZnB NPs after 8‐time recovery. **Supporting Fig. S‐28:** SEM images of CoZnB NPs after 8‐time recovery.

## Author Contributions

**Akram Ashouri**: conceptualization (lead), formal analysis (lead), funding acquisition (lead), methodology (lead), project administration (lead), resources (lead), supervision (lead), validation (lead), visualization (lead), writing – original draft (lead), writing – review & editing (lead). **Arezu Moradi**: data curation (lead), formal analysis (equal), investigation (equal), software (lead), visualization (lead), writing – original draft (equal), writing – review & editing (equal). **Behzad Nasiri**: conceptualization (supporting), formal analysis (supporting), investigation (supporting), software (lead), visualization (lead). **Somayeh Pourian**: methodology (supporting), writing – original draft (supporting). **Hossein Zamani**: investigation (supporting), visualization (supporting). **Fatemeh Rezaei**: investigation (equal), methodology (supporting). **Amin Karimizadeh**: investigation (supporting), software (supporting).

## Conflicts of Interest

The authors declare no conflicts of interest.

## Supporting information

Supplementary Material

## Data Availability

The data that support the findings of this study are available in the supplementary material of this article.
