# Supplementary material for: Bimetallic M2B Boride Nanoparticles: A Robust and Recyclable Platform for Dehydration‐Driven Condensation of Aldehydes
Source: ChemistryOpen. 2025 Dec 16;15(4):e202500596. doi: 10.1002/open.202500596 (PMC13052290; doi:10.1002/open.202500596)
Supplement: Supplementary file 1 — Supplementary Material [file OPEN-15-e202500596-s001.pdf]

Supporting Information

Bimetallic M_2B Boride Nanoparticles: A Robust and Recyclable Platform for Dehydration-Driven Condensation of Aldehydes

Akram Ashouri*, Arezu Moradi, Behzad Nasiri, Somayeh Pourian, Hossein Zamani, Fatemeh Rezaei, Amin Karimizadeh

Department of Chemistry, Faculty of Science, University of Kurdistan, 66177-15175, Sanandaj, Iran

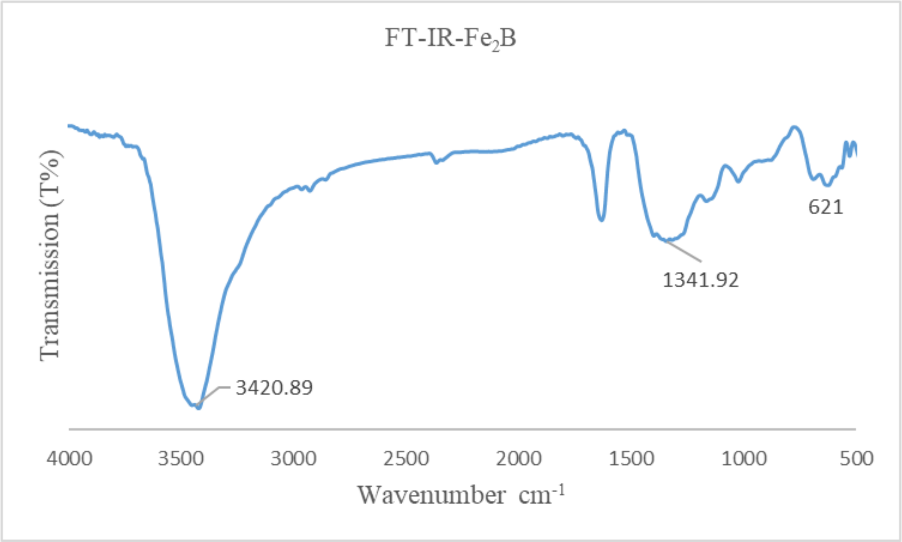

Fig. S-1: FT-IR of Fe_2B

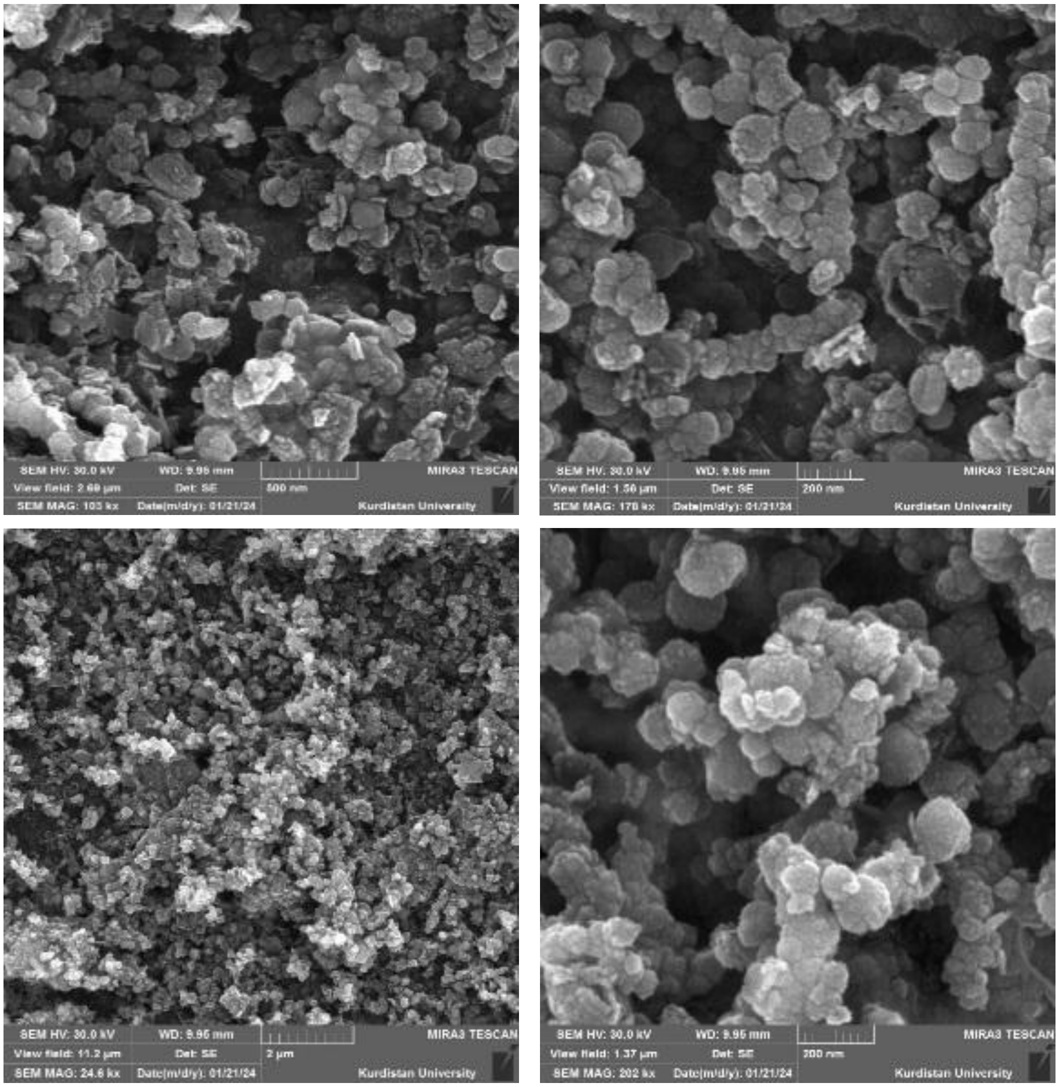

Fig. S-2: FE-SEM of Fe_2B

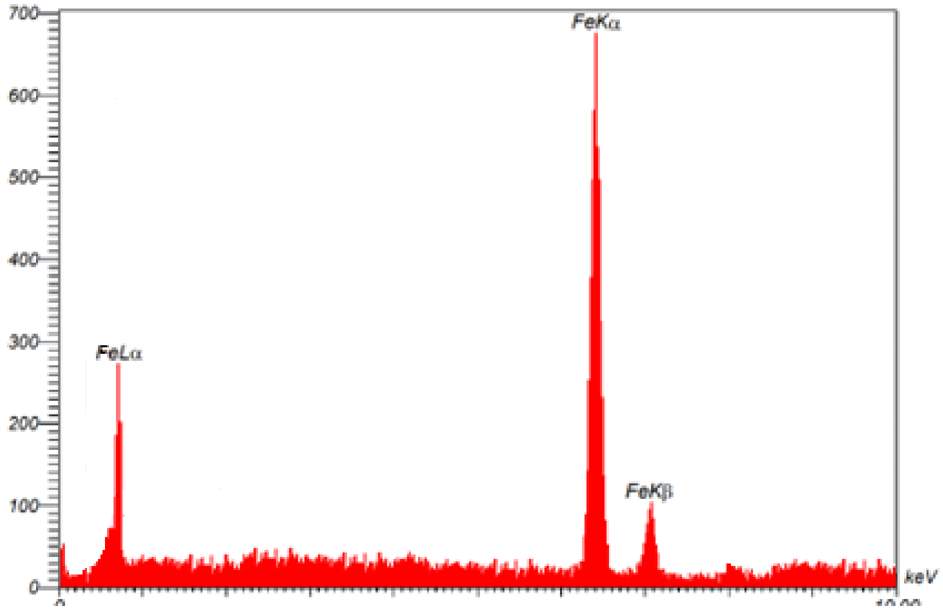

Fig. S-3: EDX of Fe_2B

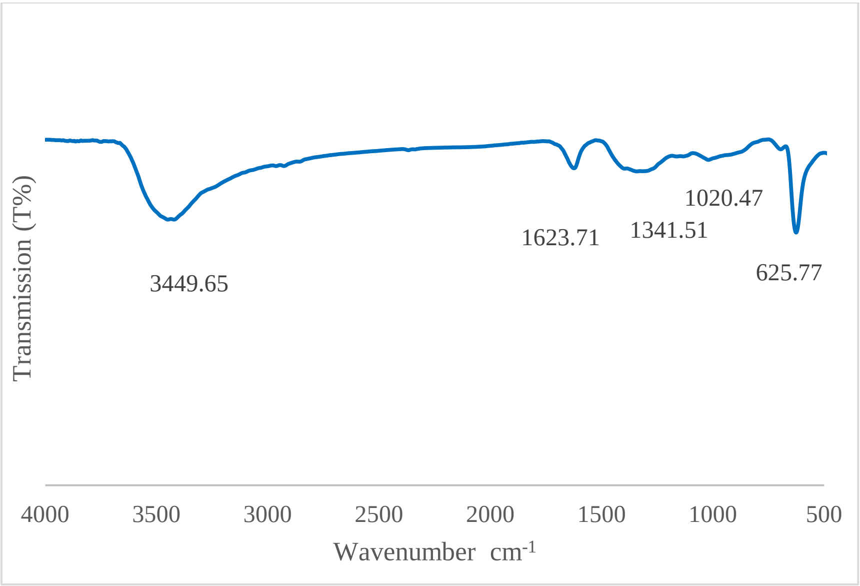

Fig. S-4: FT-IR of Cu_2B

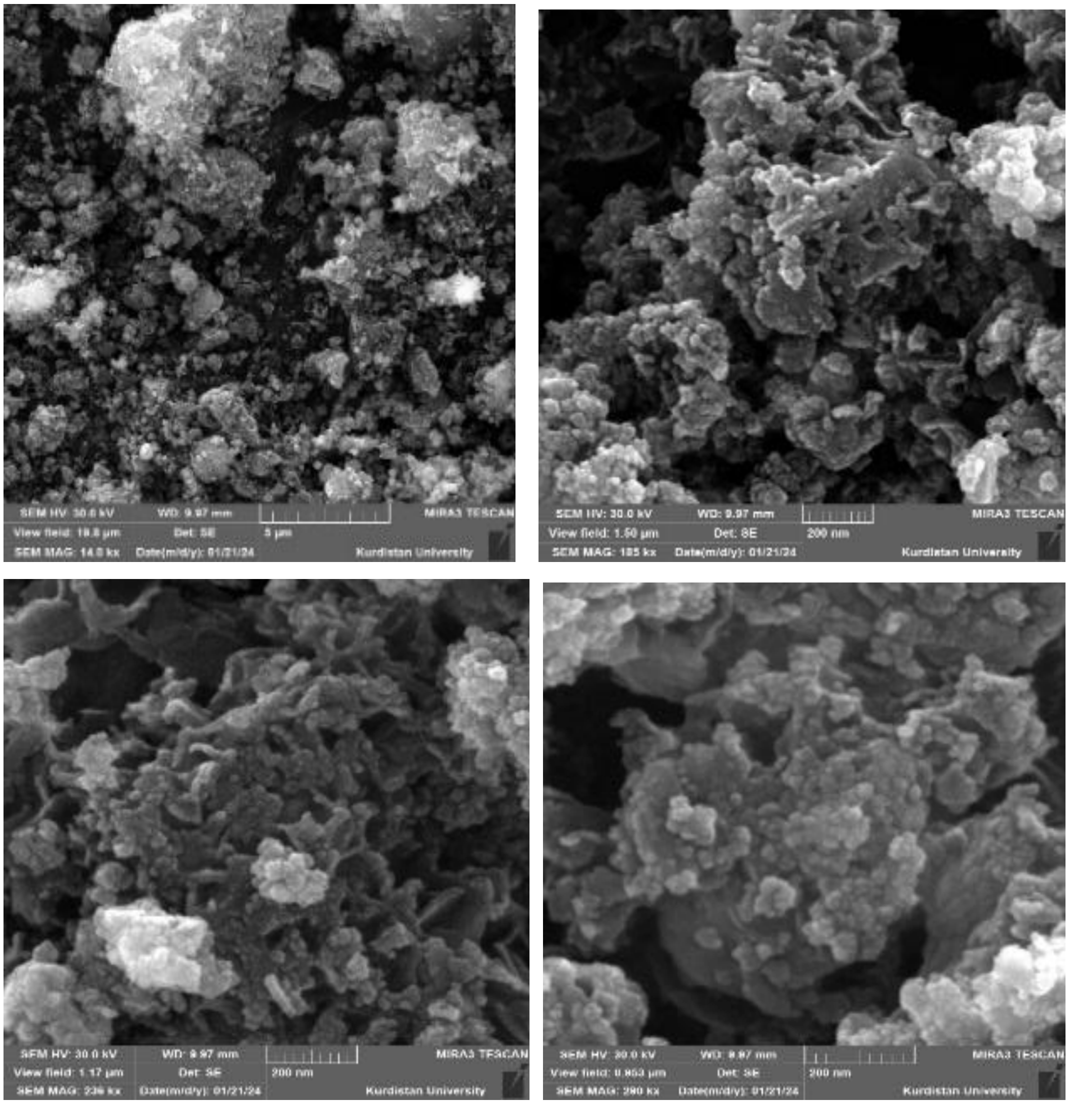

Fig. S-5: FE-SEM of Cu_2B

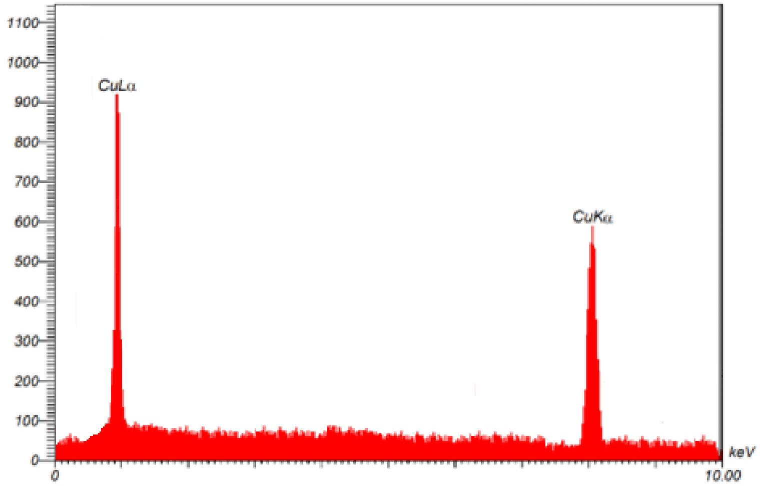

Fig. S-6: EDX of Cu_2B

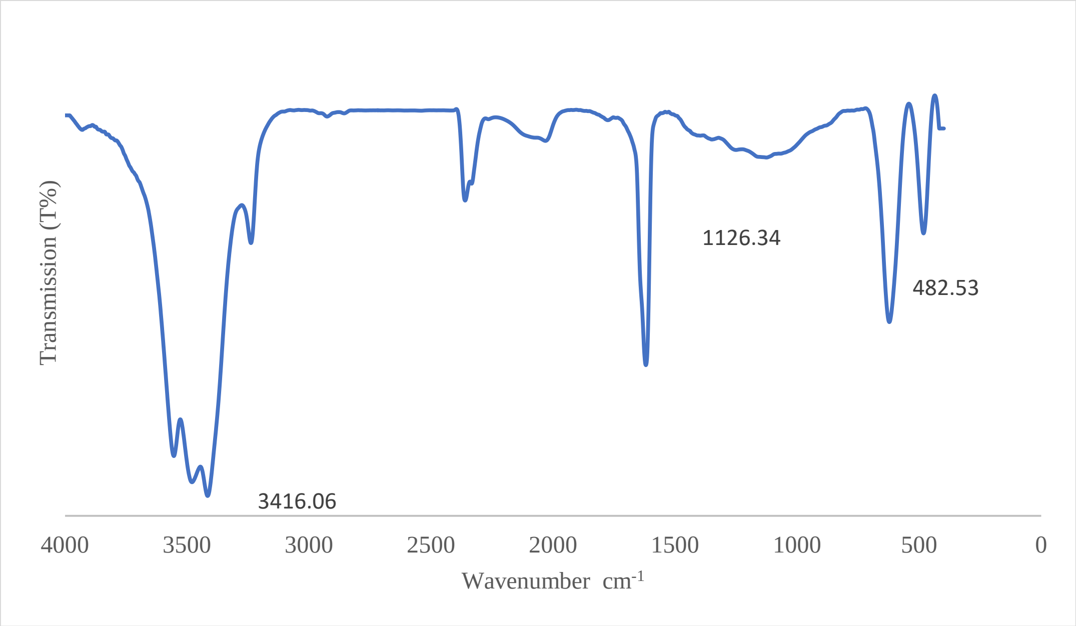

Fig. S-7: FT-IR of Co_2B

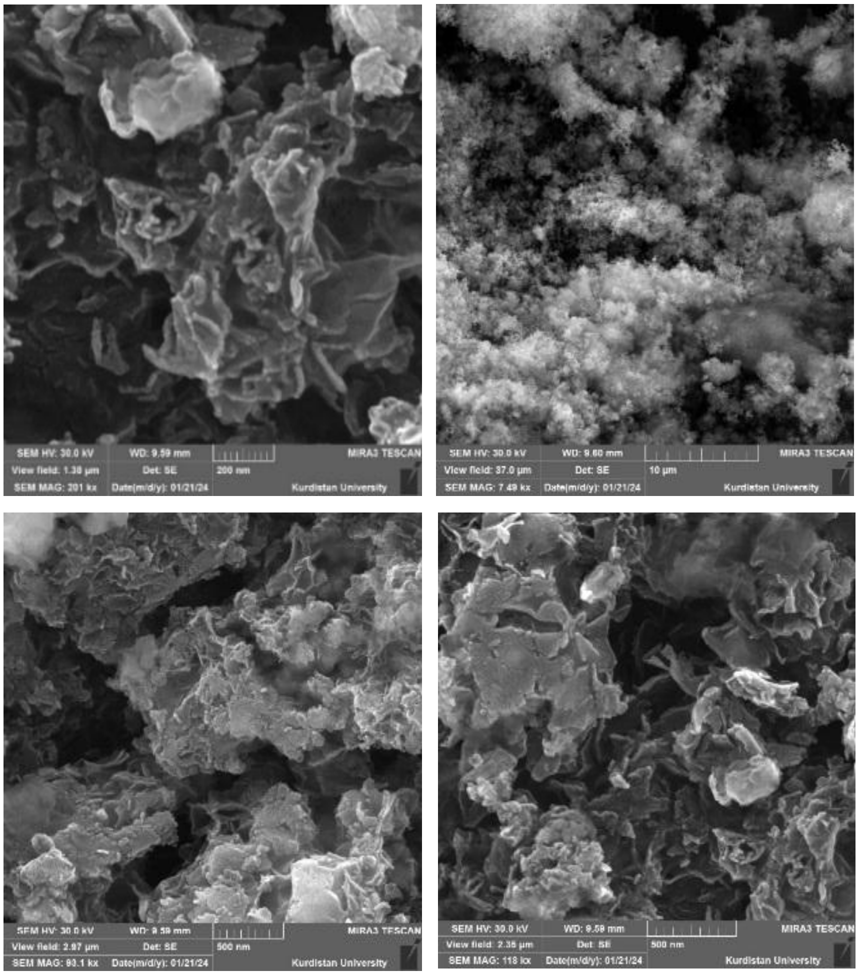

Fig. S-8: FE-SEM of Co_2B

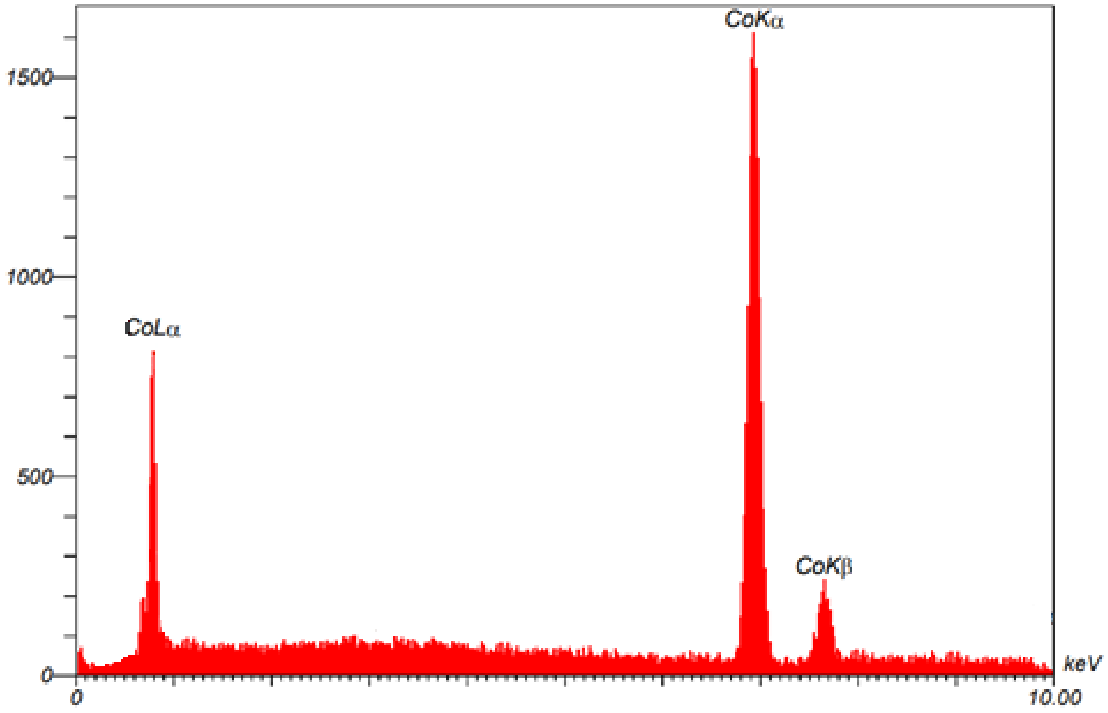

Fig. S-9: EDX of Co_2B

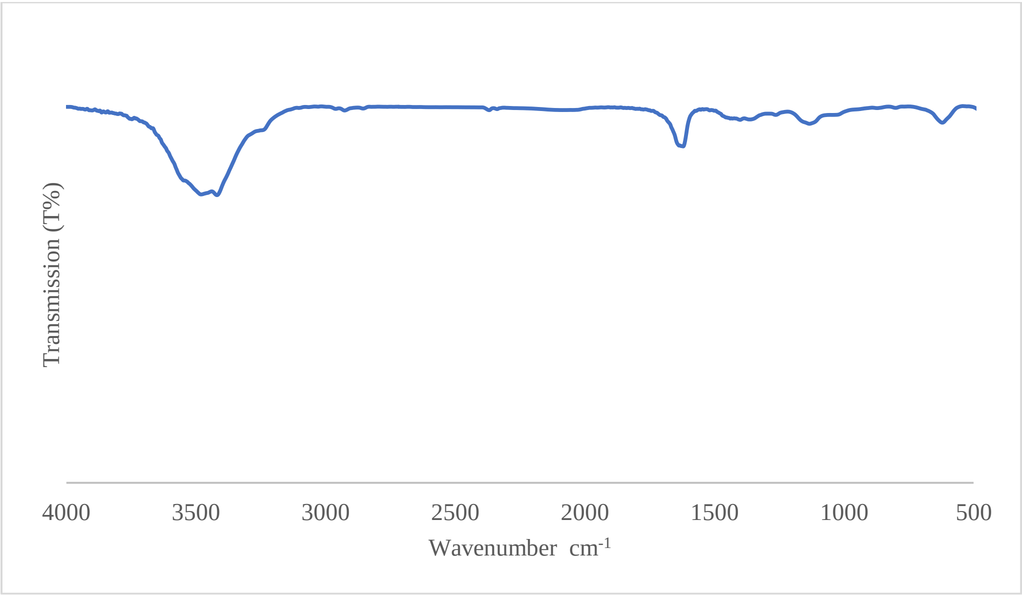

Fig. S-10: FT-IR of Zn_2B

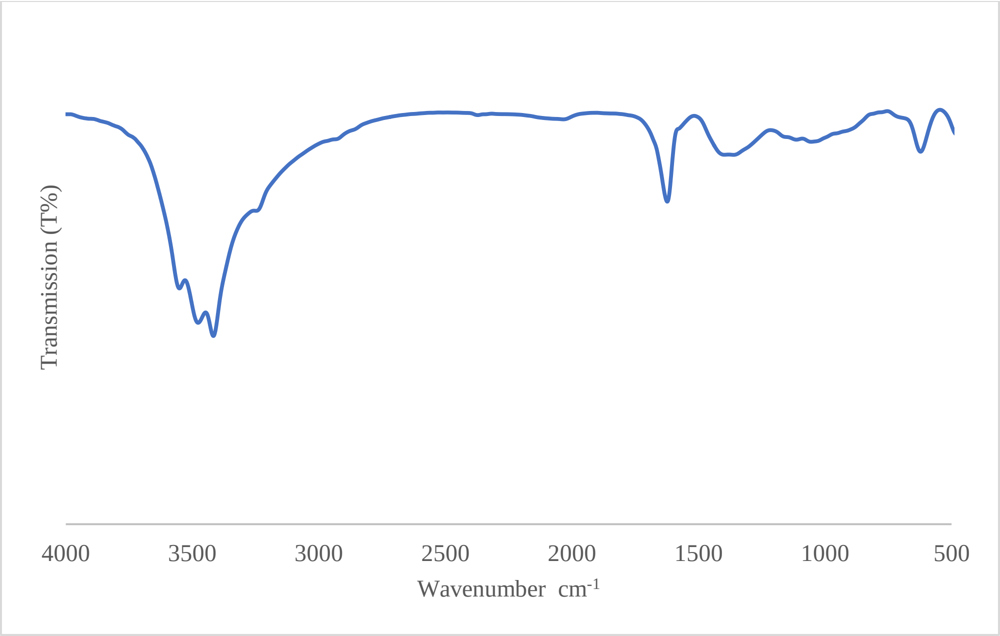

Fig. S-11: FT-IR of Zr_2B

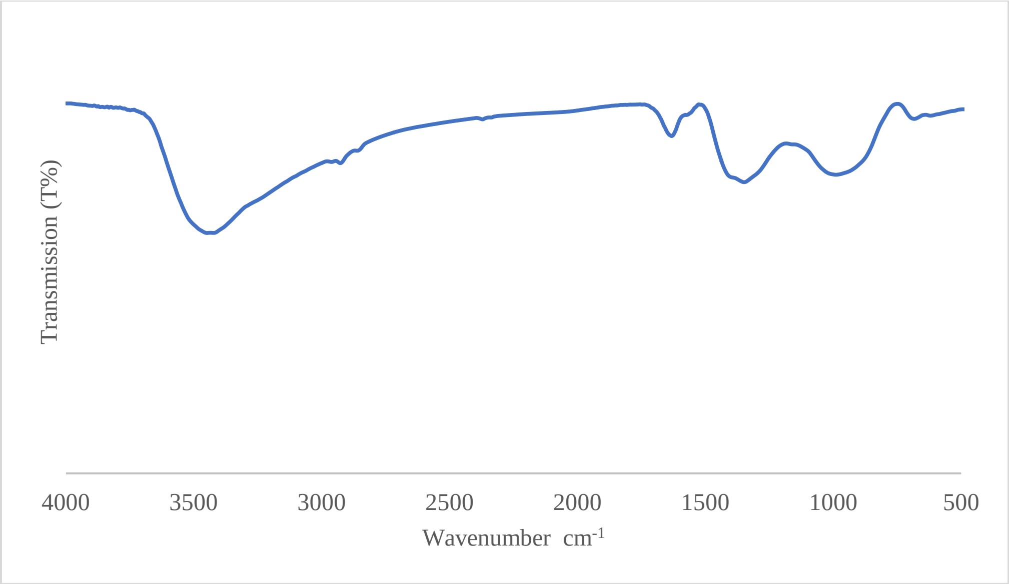

Fig. S-12: FT-IR of CoZrB

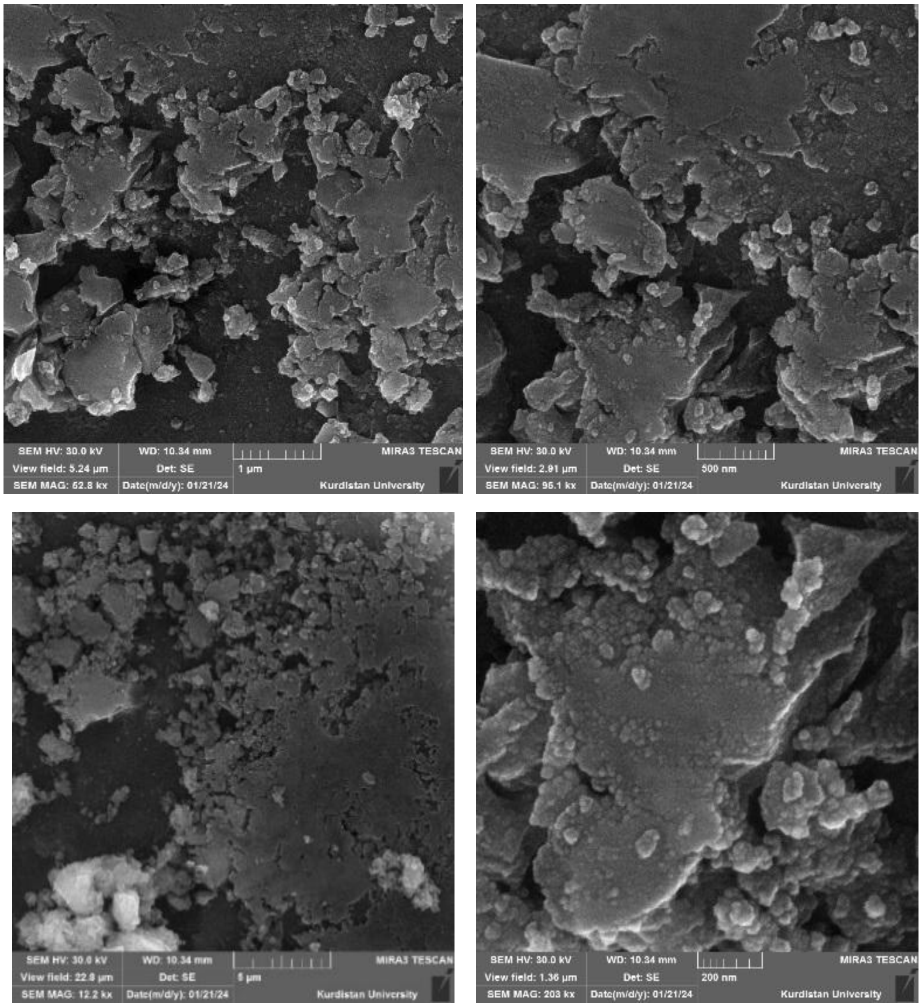

Fig. S-13: FE-SEM of CoZrB

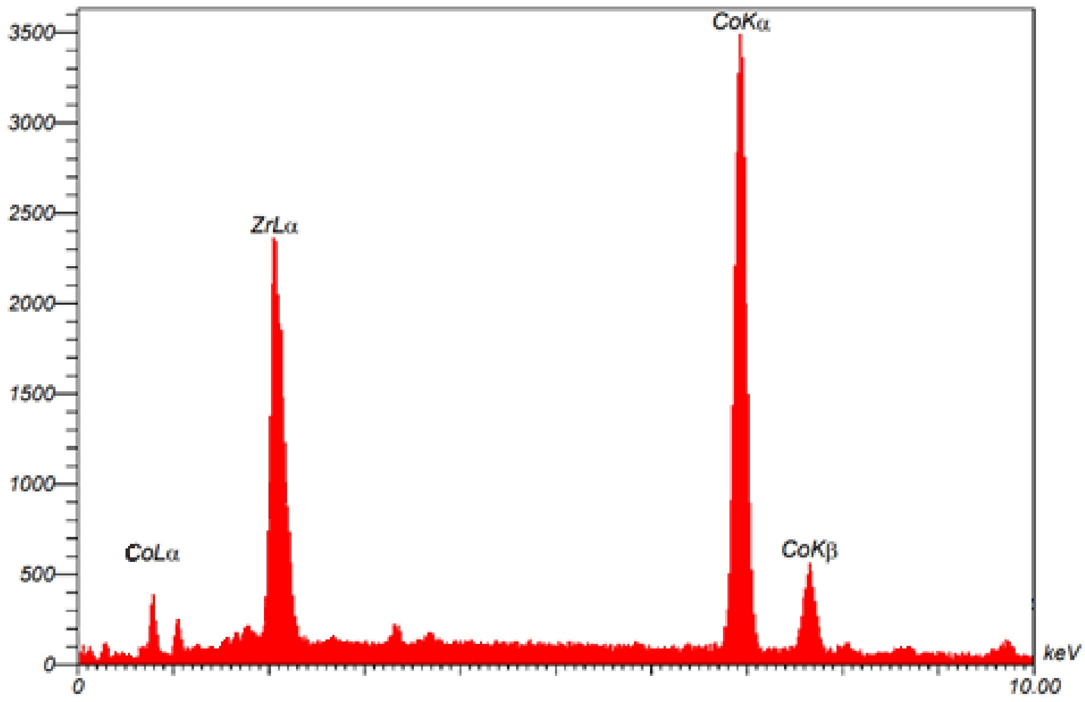

Fig. S-14: EDX of CoZrB

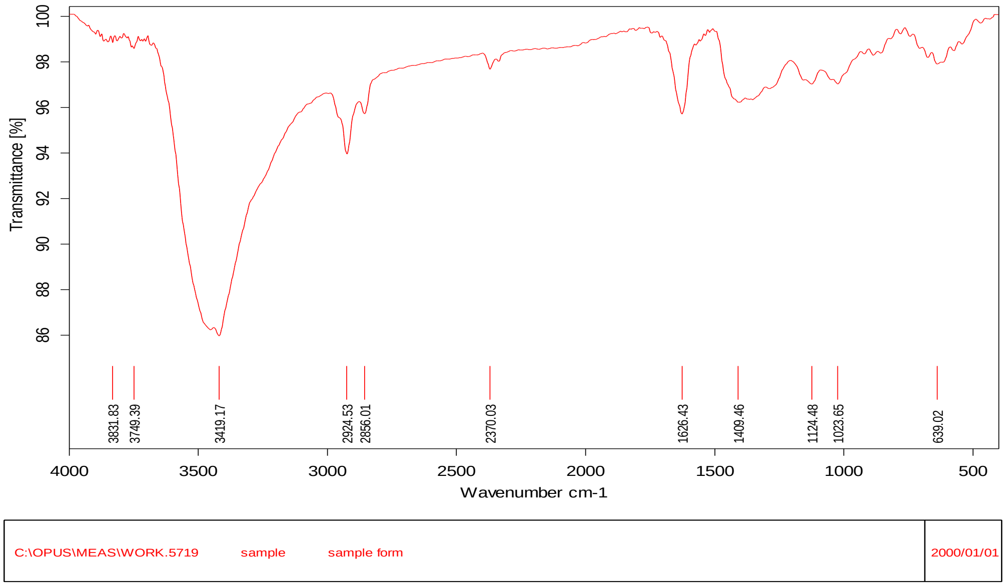

Fig. S-15: FT-IR of CoNiB

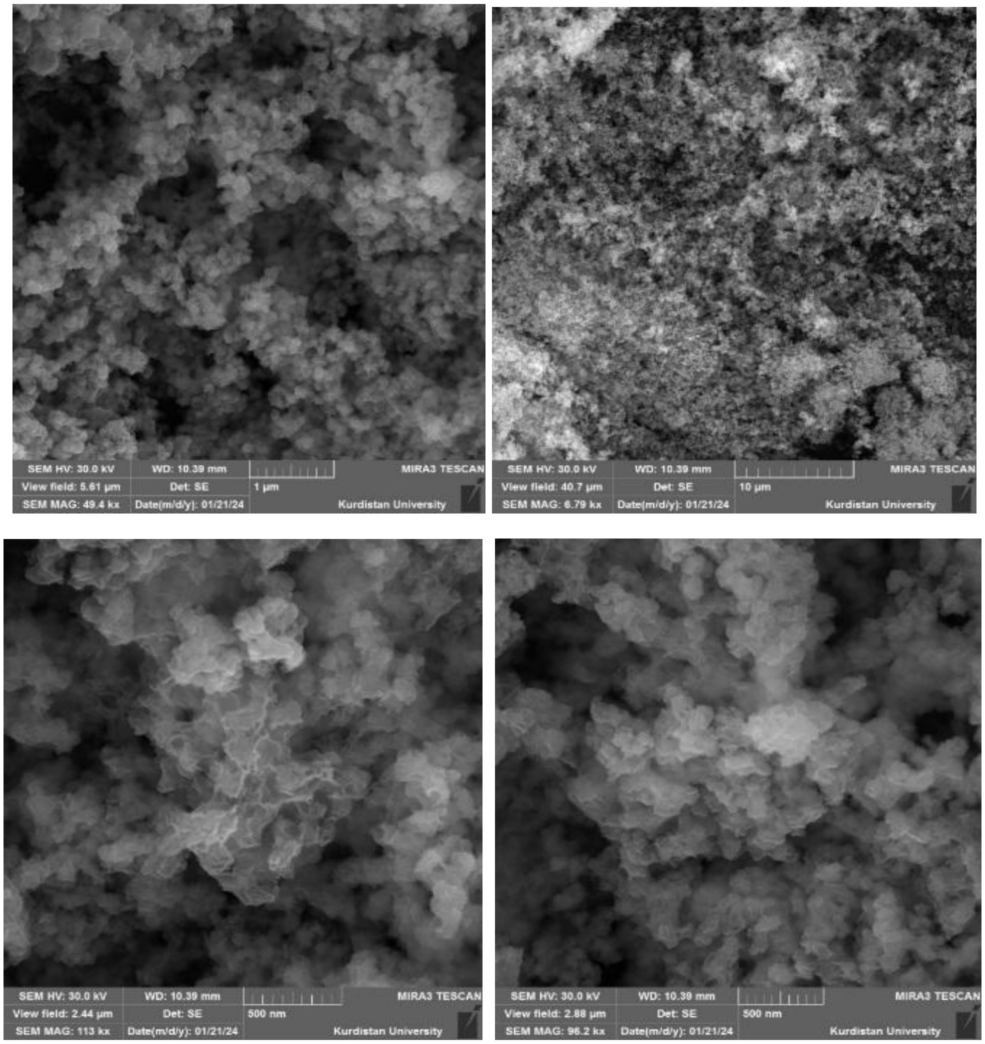

Fig. S-16: FE-SEM of CoNiB

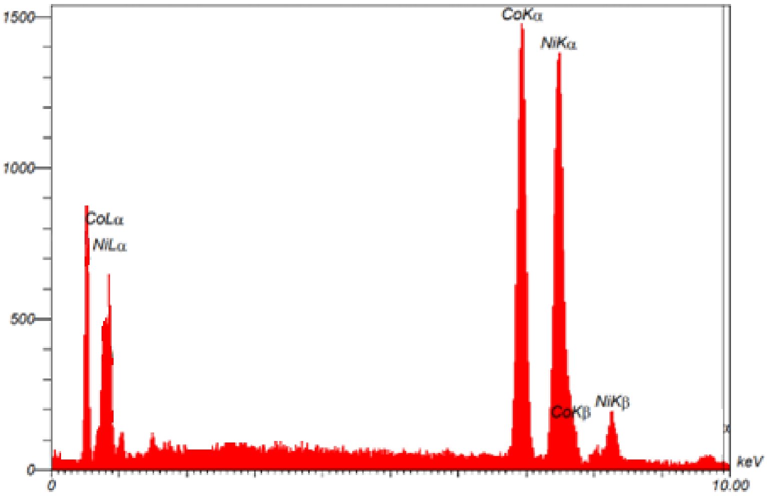

Fig. S-17: EDX of CoNiZrB

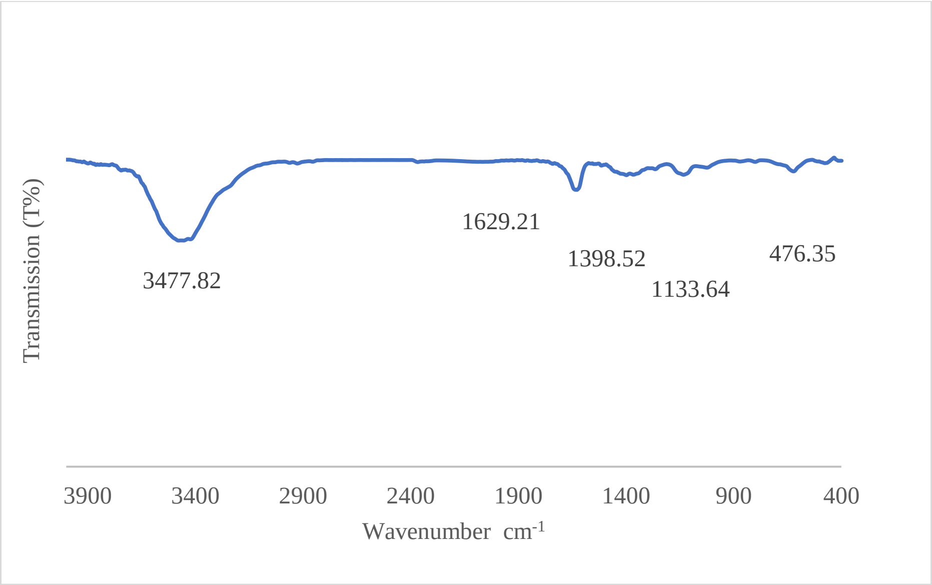

Fig. S-18: FT-IR of CuCoB

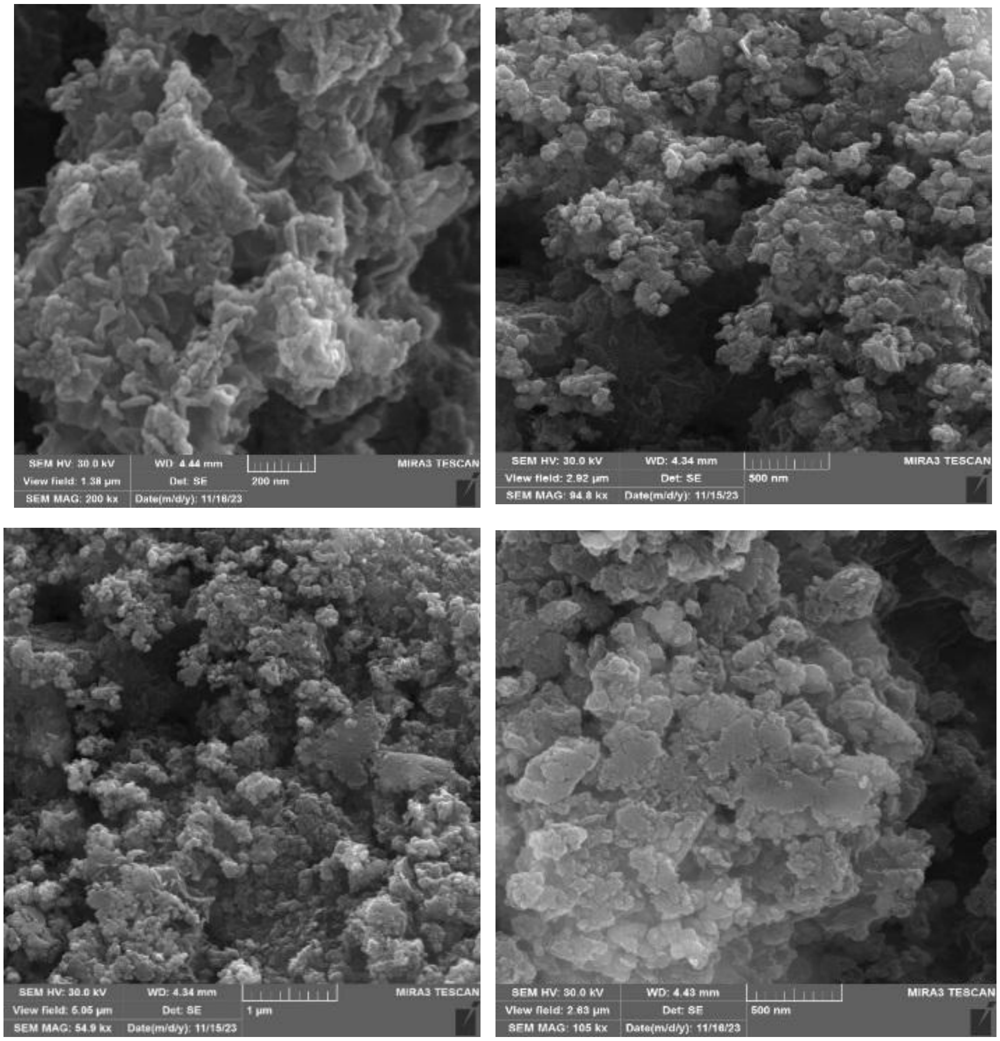

Fig. S-19: FE-SEM of CuCoB

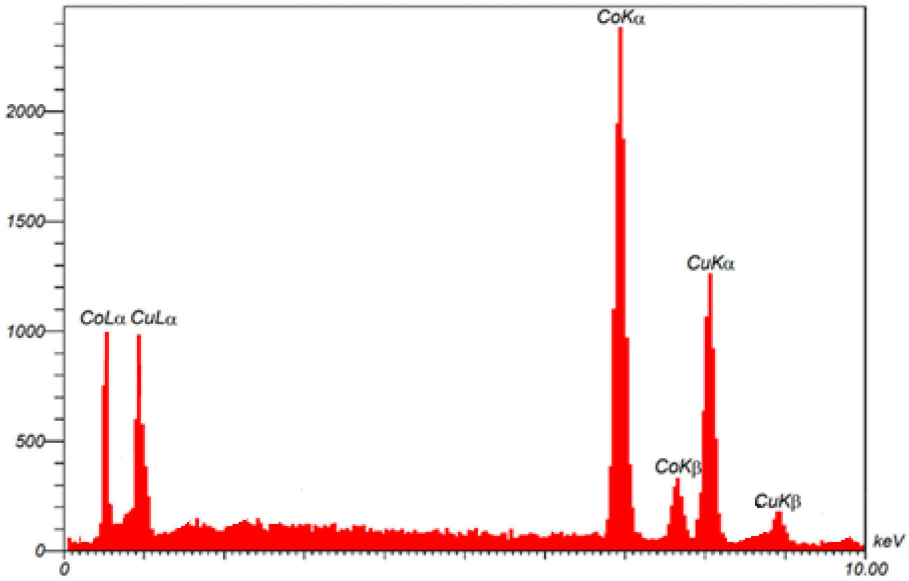

Fig. S-20: EDX of CuCoB

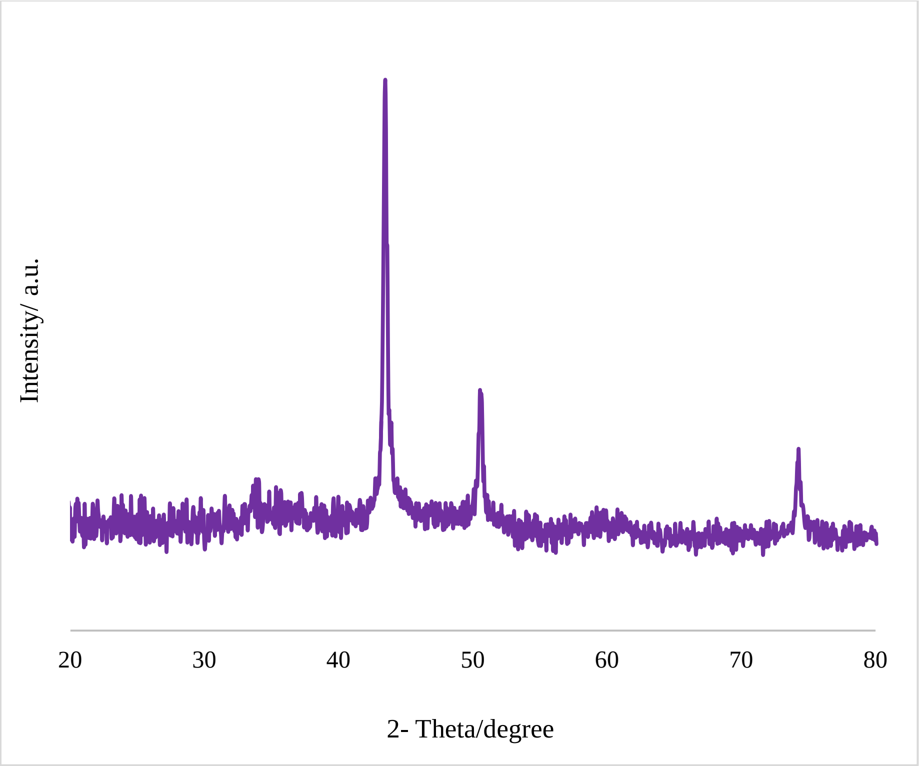

Fig. S-21: XRD of CuCoB

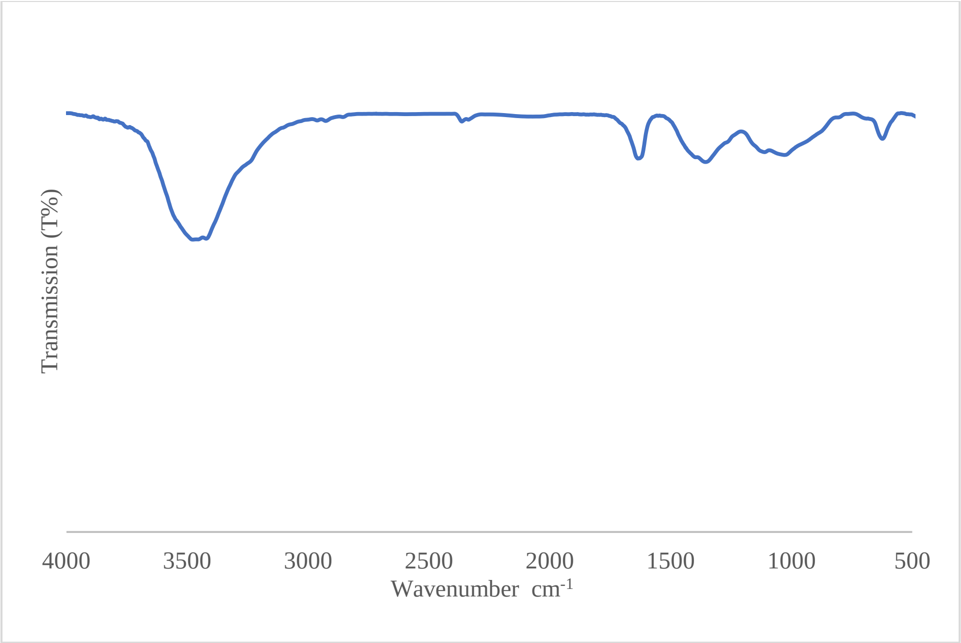

Fig. S-22: FT-IR of CuZnB

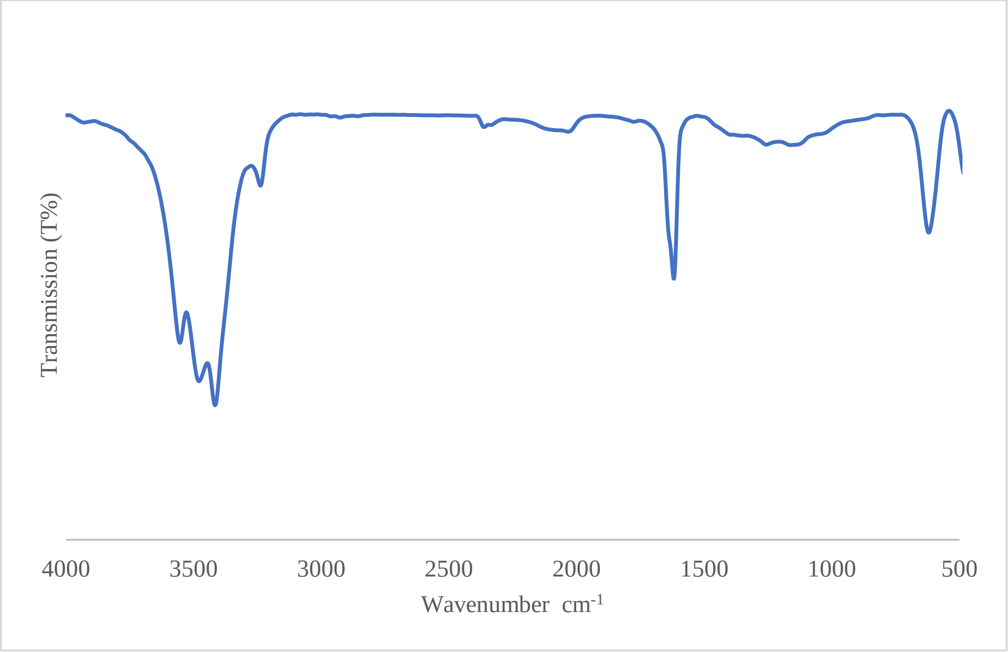

Fig. S-23: FT-IR of CuNiB

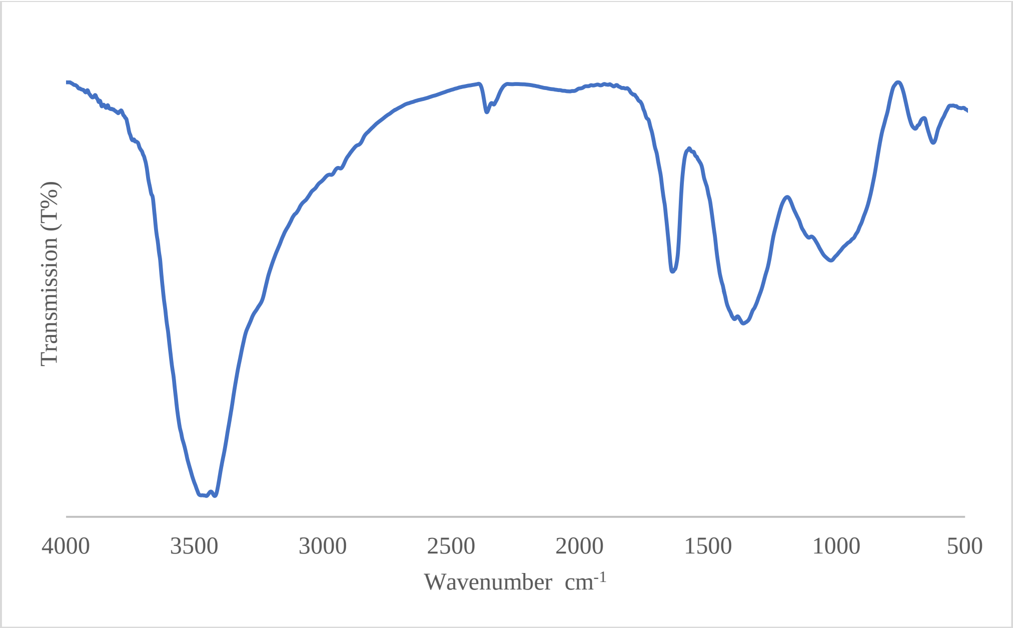

Fig. S-24: FT-IR of CuZrB

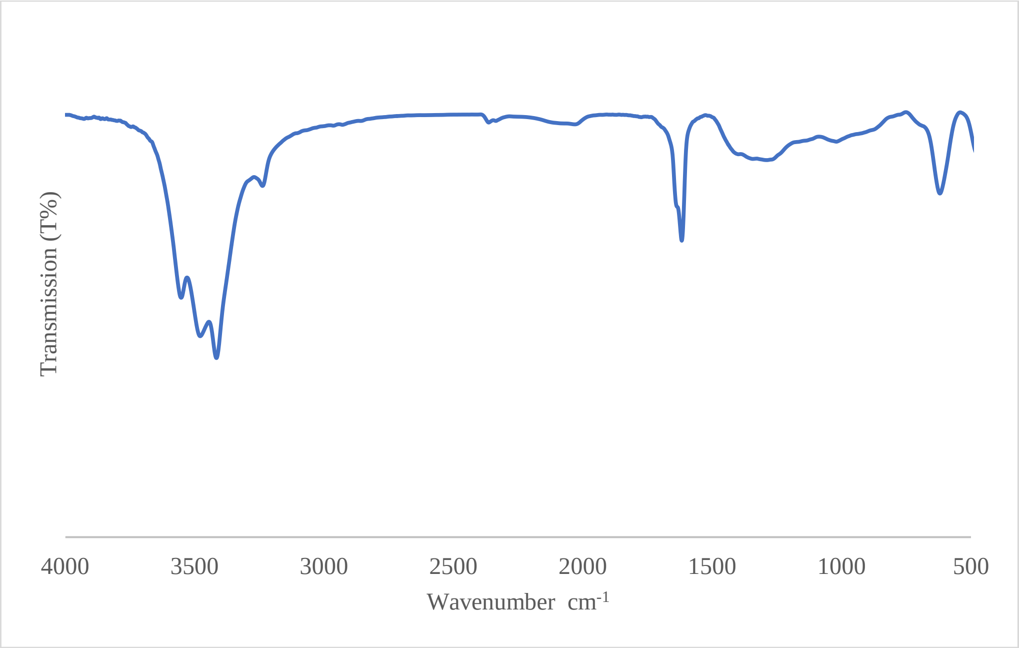

Fig. S-25: FT-IR of FeCuB

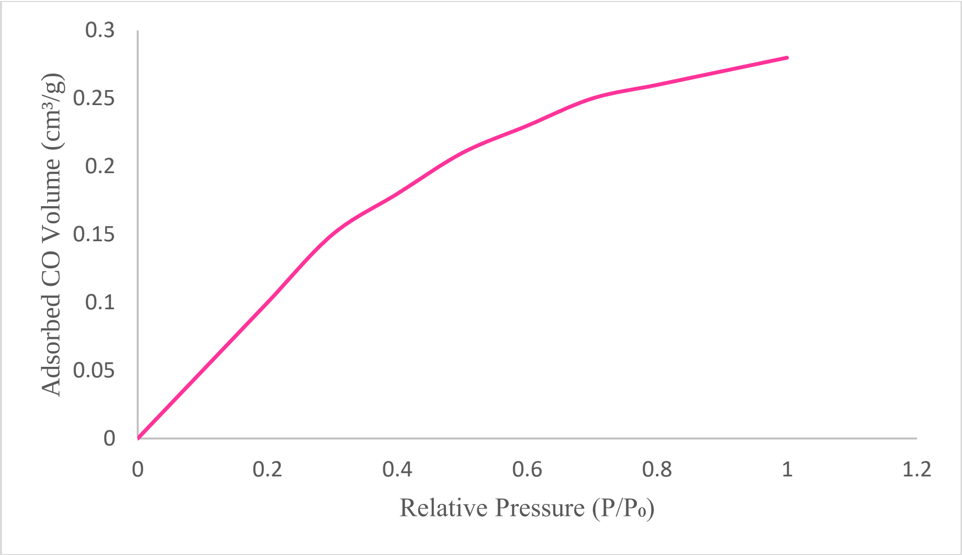

Fig. S-26: CO chemisorption isotherm of the CoZnB NPs.

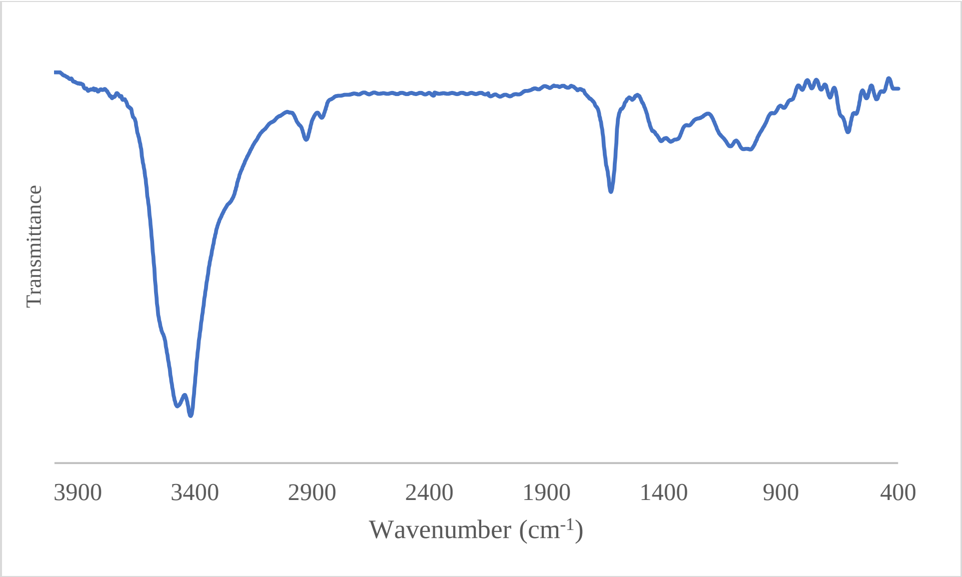

Fig. S-27: FT-IR spectrum of of CoZnB NPs after 8-time recovery.

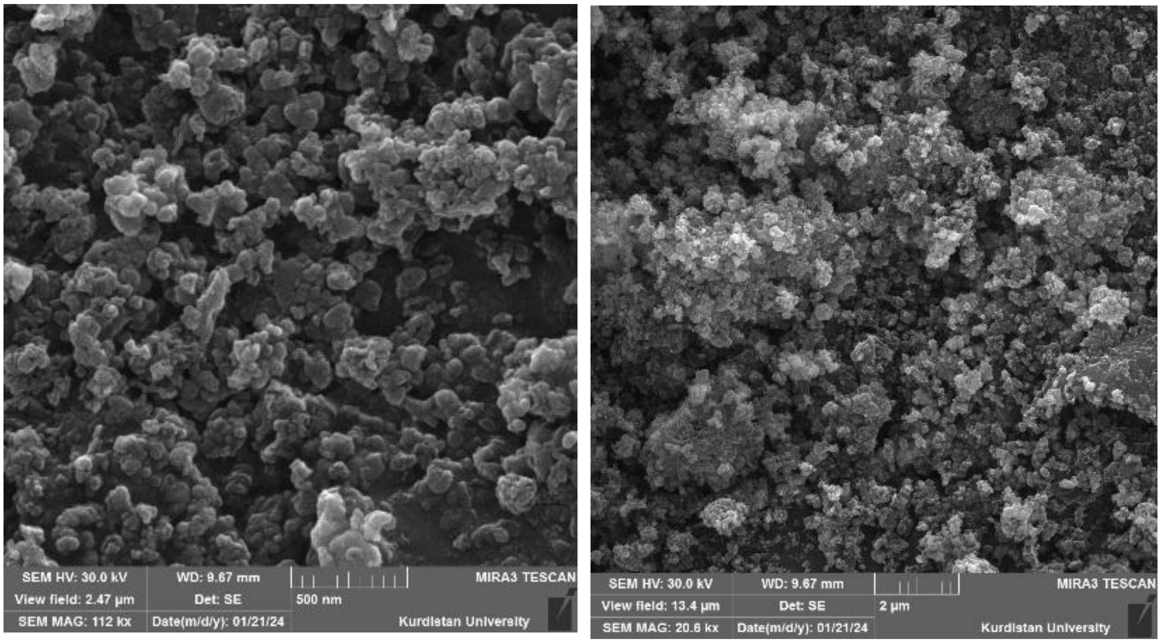

Fig. S-28: SEM images of CoZnB NPs after 8-time recovery.

NMR spectra of some products:

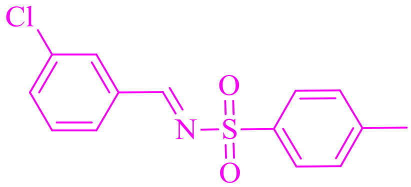

***N*-(3-chlorobenzylidene)-4-methylbenzenesulfonamide:** m.p. 90-91°C, ^{1}H-NMR (600 MHz, $CDCl_3$) δ 8.09 (s, 1 H), 7.99 (d, *J* = 7.7 Hz, 1 H), 7.81 (d, *J* = 8.5 Hz, 2 H), 7.59 (d, *J* = 8.1 Hz, 1 H), 7.43 (t, *J* = 8.1 Hz, 1 H), 7.32 (d, *J* = 8.2 Hz, 2 H), 7.26 (s, 1 H), 2.43 (s, 3 H).

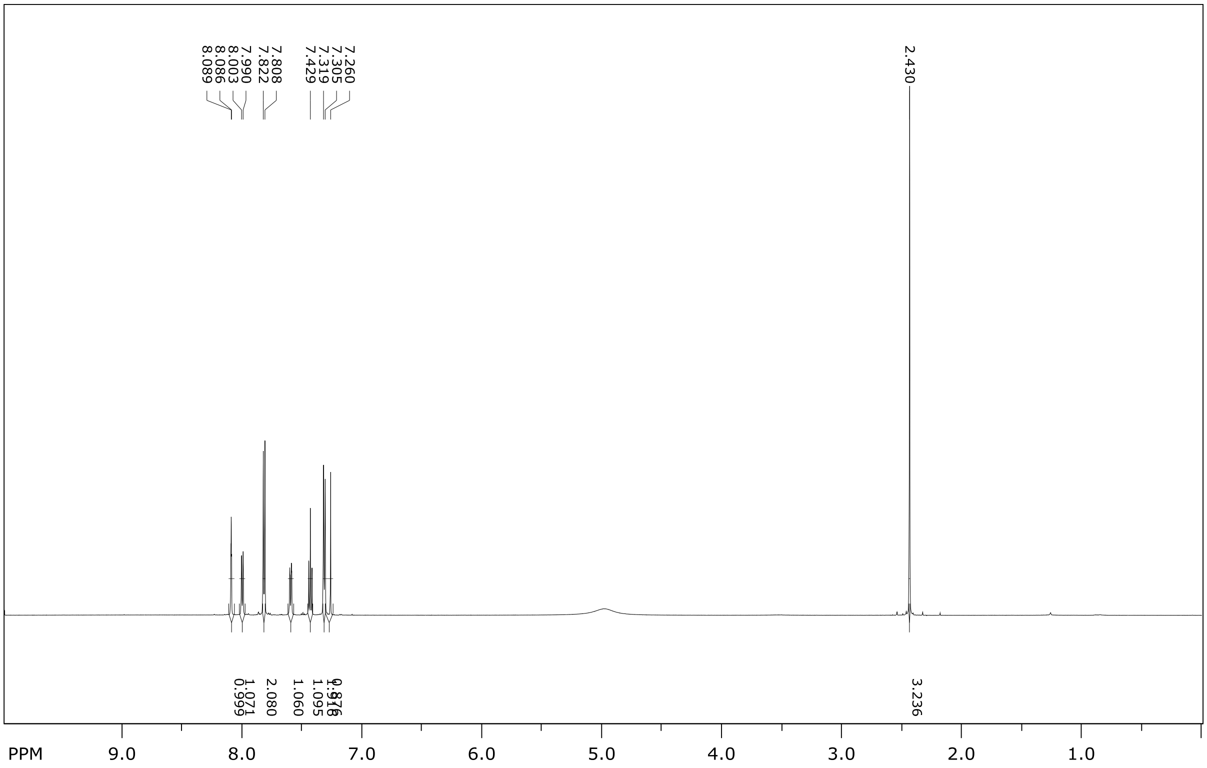

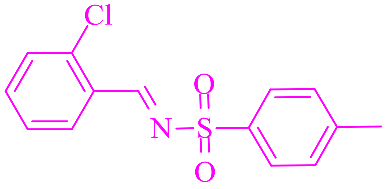

***N*-(2-chlorobenzylidene)-4-methylbenzenesulfonamide:** m.p. 131-133°C, ^{1}H-NMR (600 MHz, $CDCl_3$) δ 9.70 (s, 1 H), 7.97 (d, *J* = 8.1 Hz, 2 H), 7.85 (d, *J* = 8.2 Hz, 1 H), 6.81 (d, *J* = 8.1 Hz, 1 H), 6.75 (d, *J* = 8.1 Hz, 2 H),6.67 (d, *J* = 8.2 Hz, 2 H), 6.57 (d, *J* = 7.7 Hz, 1 H), 1.84 (s, 3 H).

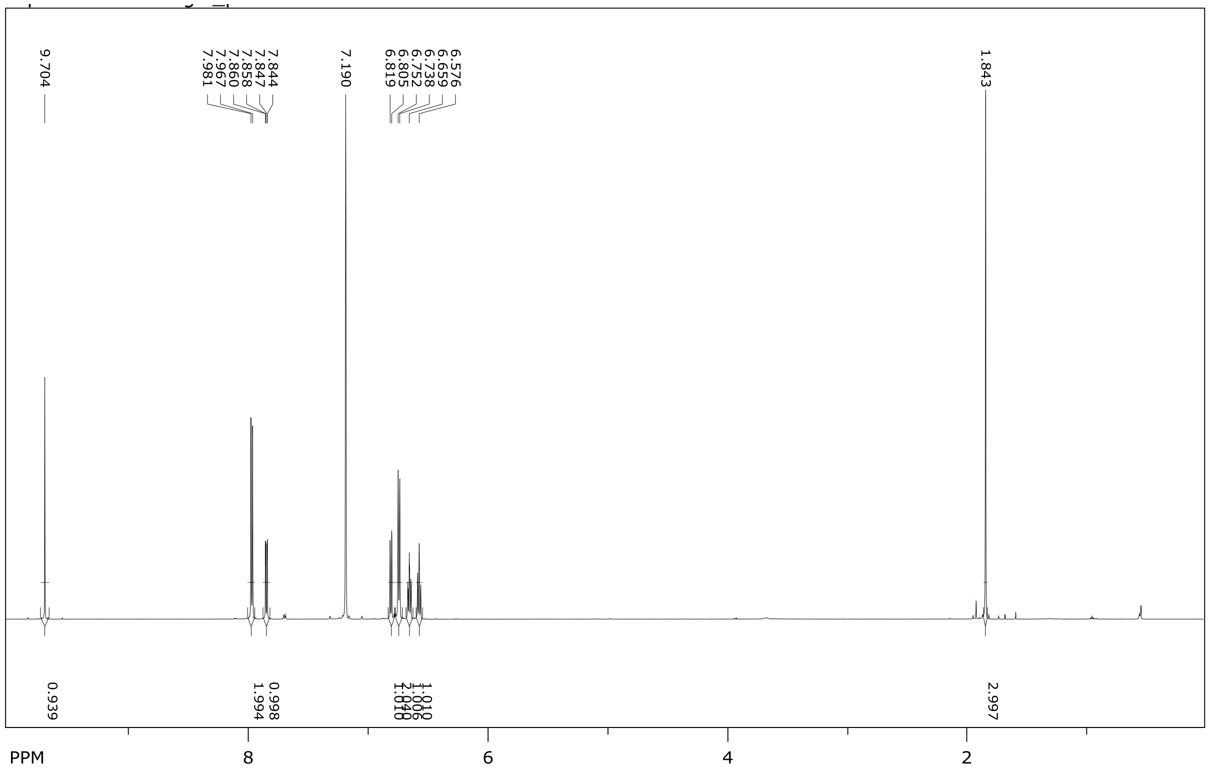

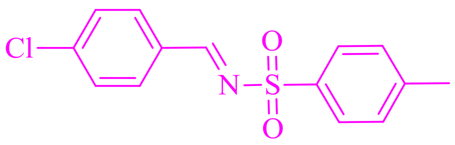

***N*-(4-chlorobenzylidene)-4-methylbenzenesulfonamide:** m.p. 165-168°C, ^{1}HNMR (500 MHz, $CDCl_3$) δ 8.99 (s, 1 H), 7.85-7.89 (m, *J* = 8.5 Hz, *J* = 8.3 Hz, 4 H), 7.46 (d, *J* = 8.5 Hz, 2 H), 7.35 (d, *J* = 8.2 Hz, 2 H), 2.43 (s, 3 H);

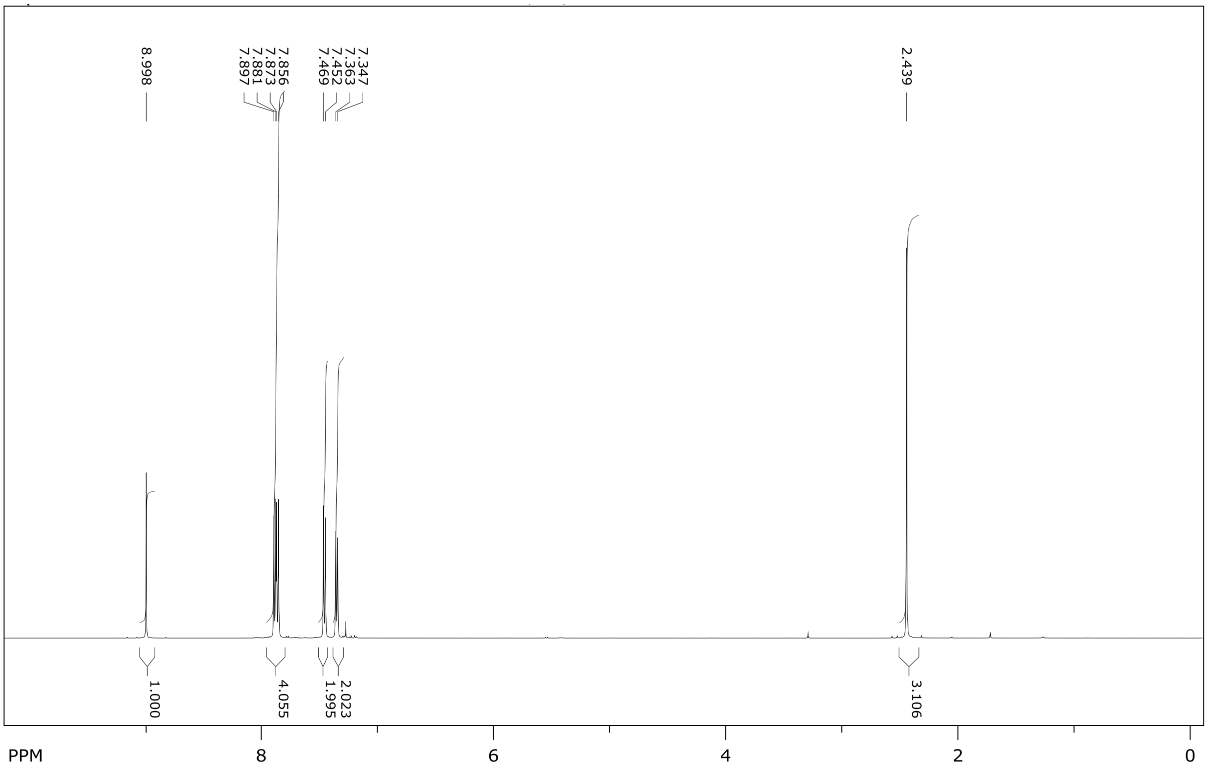

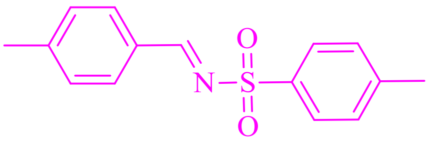

***N*-(4-methylbenzylidene)-4-methyl-benzenesulfonamide**: m.p. 126-128°C, ^{1}H NMR (500 MHz, $CDCl_3$) δ 8.99 (s, 1 H), 7.89 (d, *J* = 7.5 Hz, 2 H), 7.82 (d, *J* = 7.4 Hz, 2 H), 7.34 (d, *J* = 7.49 Hz, 2 H), 7.29 (d, *J* = 7.43 Hz, 2 H) 2.43 (s, 3 H) 2.42 (s, 3 H).

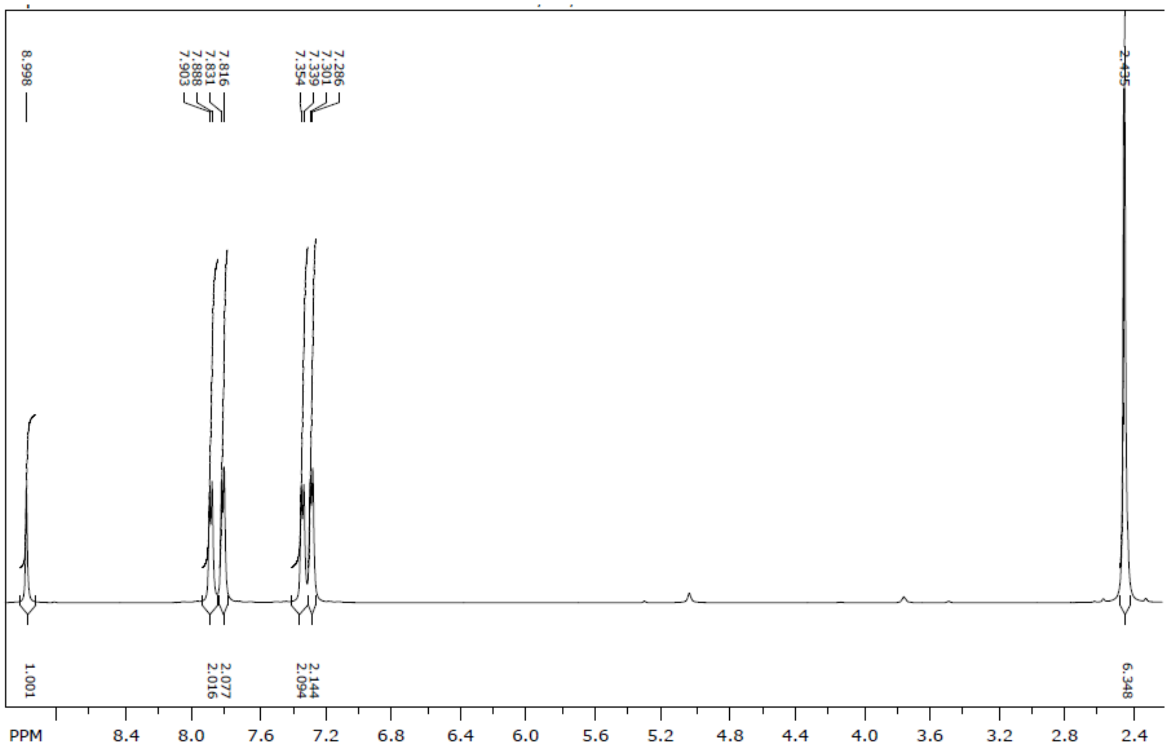

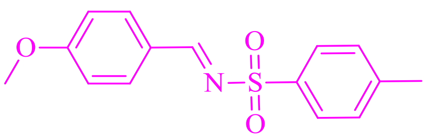

_N_-(4-Methoxybenzylidene)-4-methyl-benzenesulfonamide: m.p. 109-111°C, ^{1}H NMR (500 MHz, $CDCl_3$) δ 8.94 (s, 1 H), 7.87 (d, *J* = 8 Hz, 4 H), 7.32 (d, *J* = 7.7 Hz, 2 H), 6.96 (d, *J* = 8.2 Hz, 2 H), 3.87 (s, 3 H), 2.42 (s, 3 H).

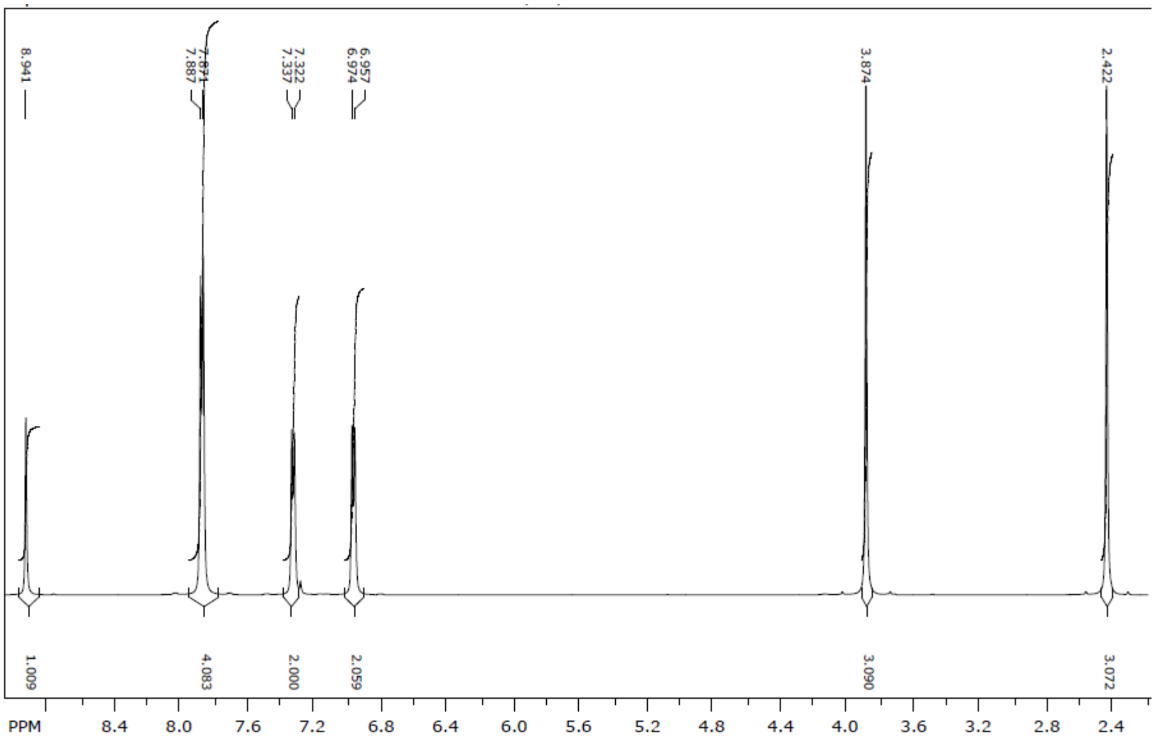

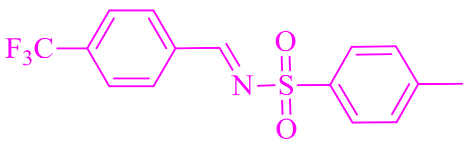

***N*-(4-(trifluoromethyl)-4-methyl-benzenesulfonamide**: m.p. 150-154°C, ^{1}H NMR (600 MHz, $CDCl_3$) δ 9.07 (s, 1 H), 8.04 (d, *J* = 8 Hz, 2 H), 7.91 (d, *J* = 8.0 Hz, 2 H), 7.75 (d, *J* = 8.5 Hz, 2 H), 7.37 (d, *J* = 8.0 Hz, 2 H), 2.45 (s, 3 H).

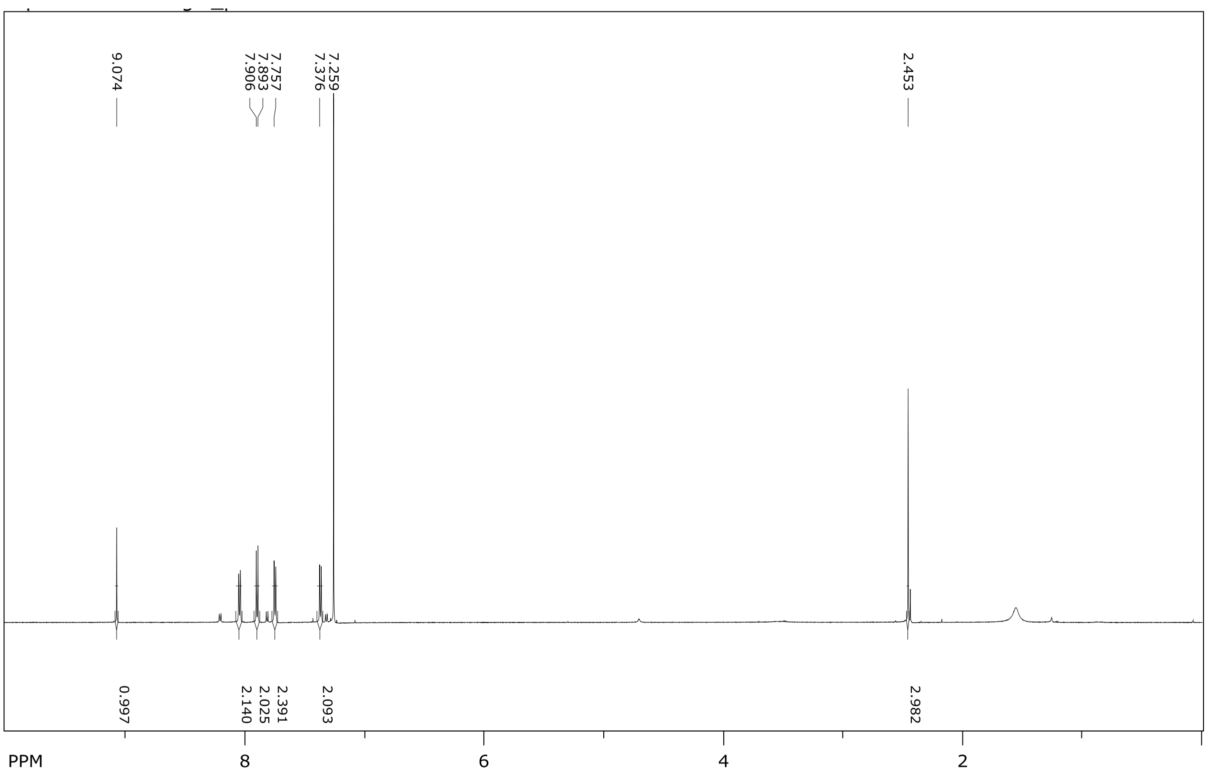

Graphical Abstract

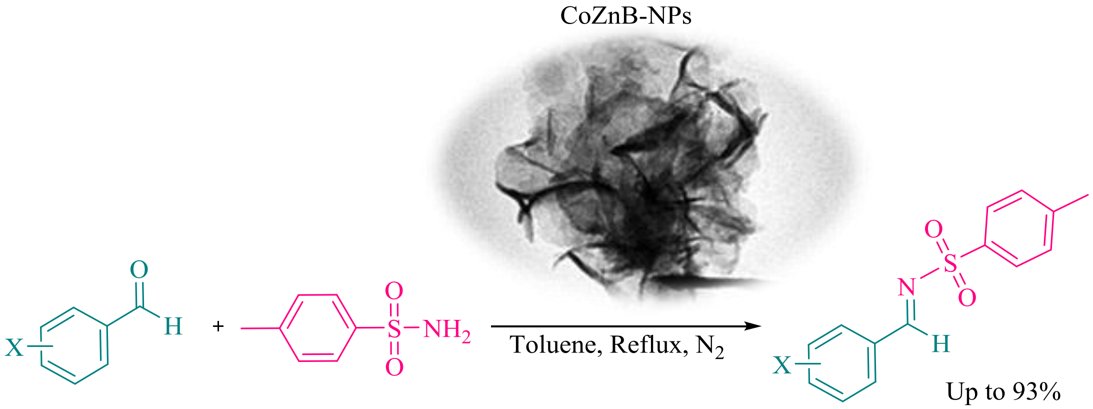

- *Bimetallic CoZnB catalyst*
- *High-yield imine synthesis*
- *Sustainable magnetic platform*
- *Mild and fast reaction*
- *Excellent recyclability*

Bimetallic M_2B Boride Nanoparticles: A Robust and Recyclable Platform for Dehydration-Driven Condensation of Aldehydes

Akram Ashouri*, Arezu Moradi, Behzad Nasiri, Somayeh Pourian, Hossein Zamani, Fatemeh Rezaei, Amin Karimizadeh
